# Rare variant discovery by deep whole-genome sequencing of 1,070 Japanese individuals

**DOI:** 10.1038/ncomms9018

**Published:** 2015-08-21

**Authors:** Masao Nagasaki, Jun Yasuda, Fumiki Katsuoka, Naoki Nariai, Kaname Kojima, Yosuke Kawai, Yumi Yamaguchi-Kabata, Junji Yokozawa, Inaho Danjoh, Sakae Saito, Yukuto Sato, Takahiro Mimori, Kaoru Tsuda, Rumiko Saito, Xiaoqing Pan, Satoshi Nishikawa, Shin Ito, Yoko Kuroki, Osamu Tanabe, Nobuo Fuse, Shinichi Kuriyama, Hideyasu Kiyomoto, Atsushi Hozawa, Naoko Minegishi, James Douglas Engel, Kengo Kinoshita, Shigeo Kure, Nobuo Yaegashi, ToMMo Japanese Reference Panel Project, Akito Tsuboi, Fuji Nagami, Hiroshi Kawame, Hiroaki Tomita, Ichiro Tsuji, Jun Nakaya, Junichi Sugawara, Kichiya Suzuki, Masahiro Kikuya, Michiaki Abe, Naoki Nakaya, Noriko Osumi, Riu Yamashita, Soichi Ogishima, Takako Takai, Teiji Tominaga, Yasuyuki Taki, Yoichi Suzuki, Masayuki Yamamoto

**Affiliations:** 1 Tohoku Medical Megabank Organization, Tohoku University, 2-1, Seiryo-machi, Aoba-ku, Sendai 980-8573, Japan; 2 Graduate School of Medicine, Tohoku University, 2-1, Seiryo-machi, Aoba-ku, Sendai 980-8575, Japan; 3 Graduate School of Information Sciences, Tohoku University, 6-3-09, Aramaki Aza-Aoba, Aoba-ku, Sendai 980-8579, Japan; 4 International Research Institute of Disaster Science, Tohoku University, 468-1, Aramaki Aza-Aoba, Aoba-ku, Sendai 980-0845, Japan; 5 Department of Cell and Developmental Biology, University of Michigan Medical School, 109 Zina Pitcher Place, Ann Arbor, Michigan 48109-2200, USA; 6 Institute of Development, Aging and Cancer, Tohoku University, 4-1, Seiryo-machi, Aoba-ku, Sendai 980-8575, Japan; 7 Graduate School of Dentistry, Tohoku University, 4-1 Seiryo-machi, Aoba-ku, Sendai 980–8575, Japan.

## Abstract

The Tohoku Medical Megabank Organization reports the whole-genome sequences of 1,070 healthy Japanese individuals and construction of a Japanese population reference panel (1KJPN). Here we identify through this high-coverage sequencing (32.4 × on average), 21.2 million, including 12 million novel, single-nucleotide variants (SNVs) at an estimated false discovery rate of <1.0%. This detailed analysis detected signatures for purifying selection on regulatory elements as well as coding regions. We also catalogue structural variants, including 3.4 million insertions and deletions, and 25,923 genic copy-number variants. The 1KJPN was effective for imputing genotypes of the Japanese population genome wide. These data demonstrate the value of high-coverage sequencing for constructing population-specific variant panels, which covers 99.0% SNVs of minor allele frequency ≥0.1%, and its value for identifying causal rare variants of complex human disease phenotypes in genetic association studies.

## Abstract (web summary)

The Tohoku Medical Megabank Organization establishes a biobank with detailed patient health care and genome information. Here the authors analyse whole-genome sequences of 1,070 Japanese individuals, allowing them to catalogue 21 million single-nucleotide variants including 12 million novel ones.

## Graphical abstract

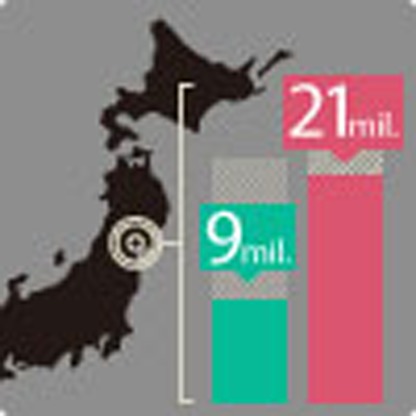

Tohoku Medical Megabank Organization (ToMMo) established a biobank that combines medical and genome information for the community medical system in the Tohoku region, located in the northeast part of Japan. We initiated the prospective genome cohort study in the region to identify genetic and environmental factors in diseases, and to enable personalized medicine based on an individual's genomic information. In the experimental design, we performed whole-genome sequencing (WGS) of 1,070 samples by PCR-free sequencing with more than 30 × coverage genome wide. This enabled us to identify very rare as well as novel single-nucleotide variants (SNVs), which was impossible to find by single-nucleotide polymorphism (SNP) microarrays or are difficult to find by low coverage sequencing on the same sample size. Notably, all sequencing and bioinformatics analyses were conducted using the same protocols in a single institute, allowing stringent control over systematic errors that might arise from using different equipment, protocols or bioinformatics pipelines.

Since the International Human Genome Project was completed^1^, a great deal of effort has been devoted to discovering, cataloguing and haplotyping common nucleotide sequence variants in populations by targeted sequencing and SNP arrays, such as in the International HapMap Project^2^. The knowledge of these variants enabled genome-wide association studies (GWASs), through which many SNPs associated with human traits and diseases have been discovered^3^,^4^ under the assumption of common-disease/common-variants hypothesis^5^. However, these identified SNPs can only explain a small fraction of genotype–phenotype relationships underlying the problem of ‘missing heritability^6^'. Recent GWAS conducted on a large number of case and control samples revealed that lower-frequency variants contribute to a substantial fraction of the heritability of common diseases, such as type 2 diabetes^7^ and cancers^8^. The 1000 Genomes Project (1KGP) has catalogued human genetic variations at minor allele frequency (MAF) >1% through WGS from multiple ethnic groups^9^. However, low frequency (0.5%<MAF≤5%), rare (0.1%<MAF≤0.5%) or very-rare (MAF≤0.1%) variants have not been thoroughly catalogued, in comparison with common variants (MAF>5%) because the existence of lower-frequency variants is population-specific^9^. Targeted high-coverage sequencing has been conducted to detect very-rare variants in the coding sequences of European Americans and African Americans^10^,^11^, while, in contrast, the majority of rare and very-rare variants in intergenic regions have not yet been discovered. Since the ENCODE project showed that a substantial fraction of noncoding regions (80.4%) are biochemically active^12^, these data suggest that these regions may also be associated with phenotypes or diseases.

Structural variants (SVs), including copy-number variants (CNVs), have also been catalogued in several studies^13^,^14^. In addition, a number of studies have revealed associations of SVs with occurrence of disease, including autism^15^, schizophrenia^16^ and Crohn's disease^17^. CNVs may also explain traits of populations, such as dietary habits of agricultural societies and hunter–gatherers^18^, or drug responses^19^. For identifying SVs, a middle-coverage sequencing strategy (∼13 × ) with 250 parent–offspring families by the Genome of the Netherlands succeeded in extending the catalogue of deletions from 20 to 100 bp compared with the 1KGP data set^20^. As high-coverage WGS is becoming less expensive, it is now feasible to detect lower-frequency SVs as well as SNVs^21^,^22^,^23^ in specific target populations^24^.

From the identified SNVs, we construct a reference panel of 1,070 Japanese individuals (1KJPN), including some very-rare SNVs. The 1KJPN cohort provides unique insights into the landscape of functional variations, especially in noncoding regions. We demonstrate here that 1KJPN is useful for genotype imputation for the Japanese population. In this analysis, a functional variant associated with Moyamoya disease (MMD) was identified through the imputed genotypes based on 1KJPN.

## Results

### Data processing and variant discovery

From the collected DNA samples in the ToMMo biobank, we selected 1,344 candidates for constructing 1KJPN, considering the traceability of participants' information, and the quality and abundance of DNA samples for SNP array genotyping and WGS analysis. All participants provided written informed consent, and all DNA samples and personal information were analysed anonymously. All the DNA samples were genotyped with Illumina HumanOmni2.5–8 BeadChip (Omni2.5). Among the genotyped samples, 1,070 samples were then selected by filtering out close relatives and outliers (Supplementary Fig. 1).

**Figure 1:**
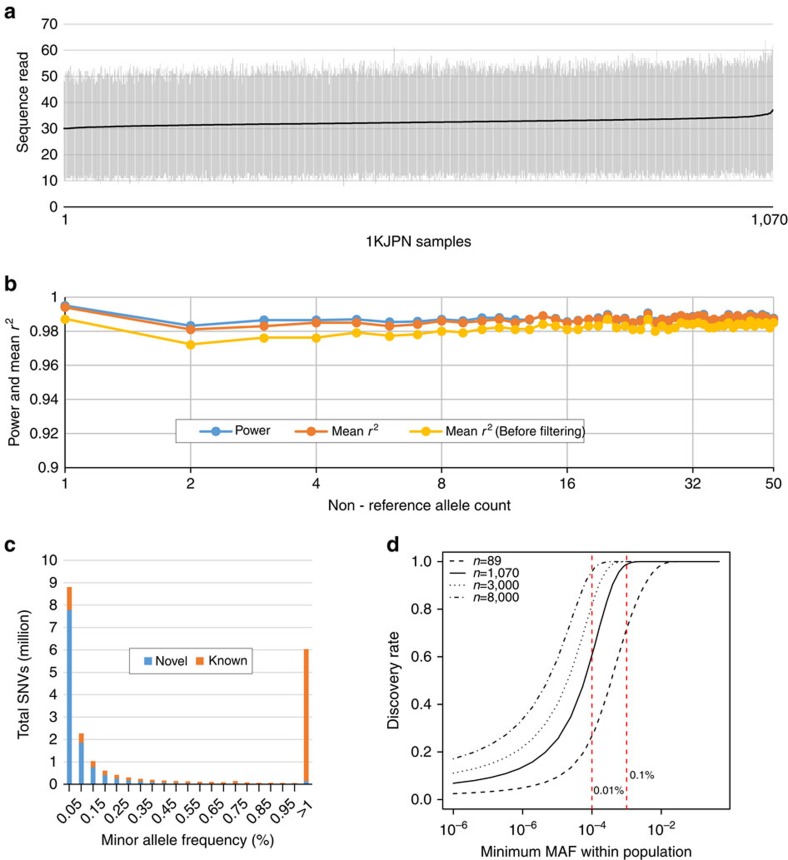
SNVs in 1KJPN. (**a**) Statistics on read depth in 1KJPN. The vertical bars indicate the minimum and maximum depth of the number of sequence reads on each individual after filtering. They were sorted according to the average sequenced read depth (the black line). (**b**) The plot shows the power to detect SNVs (blue) of the confidence SNVs and the mean *r*^2^ values before (yellow) and after (orange) filtering with SNP array data for the same sample on non-reference allele counts ranging from 1 to 50. The *r*^2^ between genotypes from the SNVs in 1KJPN and the SNP array data is given by the squared Pearson correlation. (**c**) The numbers of novel and known SNVs in each MAF bin. The novel SNV frequency begins to dominate for lower MAFs. (**d**) The rate of variant discovery by minimum MAF in the 1KJPN population. The rates of variant discovery in our sequencing strategy were plotted against minimum MAF in the 1KJPN population by different sampling size. The distribution of population MAF was estimated on the basis of the demographic model shown in Supplementary Fig. 3.

PCR bias is one of the major sources of sequencing error^25^. Hence, the selected 1,070 samples were sequenced by Illumina HiSeq 2500 using the latest PCR-free protocol (162 bp paired-end reads and 550 bp insert size, improving the accuracy for detecting SVs^26^; Fig. 1a and Methods). An in-house density check protocol was employed before sequencing^27^, and subsequent data quality-control (QC) was performed with originally developed software^28^ named SUGAR to maximize the quality and throughput of each experiment. In total, 100.4 trillion bases of DNA sequence reads were generated (Table 1). The sequence reads were aligned to the human reference genome (GRCh37/hg19) with decoy sequences (hs37d5), and then variants were called by several computational algorithms (see Methods). This strategy led to the discovery of 29.6 million SNVs (the high-sensitive SNVs), 1.97 million short deletions (72.6% novel), 1.38 million short insertions (<100 bp; 75.0% novel), 47,343 large deletions and 9,354 large insertions (equal to or longer than 100 bp) in autosomes (Table 1).

To obtain reliable SNV calls, we applied multiple filtering steps (Supplementary Fig. 2), including the depth of coverage of reads (Fig. 1a), software-derived biases, departure from Hardy–Weinberg equilibrium (HWE) and complexity of genomic regions around variants (Methods and Supplementary Table 1). The performance of genotype calls in the high-confidence SNVs was improved after filtering (Fig. 1b). Consequently, we obtained a 21.2-million SNV call set, which hereafter referred to as the high-confidence SNVs (56.6% are novel; Table 1 and Fig. 1c). The high novel SNV ratio is consistent with a previous observation that rare variants tend to be population-specific^20^. The false discovery rate (FDR) of the high-confidence SNVs was confirmed using several different experimental technologies (see Methods); the FDR for SNVs, deletions and insertions were 0% (0 out of 174; confidence interval (CI) 0.0–1.10%), 0% (0 out of 32; CI 0.0–5.78%) and 3.85% (1 out of 22; CI 0.49–19.34%), respectively (Supplementary Table 2). We further conducted validation experiments for novel SNVs using a custom-designed Illumina SNP array. Combined with the genotyping results obtained with Omni2.5, the overall FDR was 0.8% with CI 0.63–0.97% (Supplementary Table 3 and Methods). It is important to note that the estimates of FDRs were not strongly affected by MAF, indicating that the discoveries of novel variants in this study are fairly robust with respect to the allele frequency.

### Estimation of variant discovery rate

We estimated the rate of variant discovery with the sample size of 1,070. Because the distribution of allele frequency in a population is affected by underlying demographic history^29^,^30^, we inferred the demographic model of 1KJPN population from the site frequency spectrum (SFS) constructed from the intergenic regions (Supplementary Fig. 3a). As expected from excess in rare variants, the population of 1KJPN has experienced recent population expansion (Supplementary Fig. 3b), which is consistent with previous studies^10^,^30^. This demographic model is used for the calculation of the variant discovery rate (Fig. 1d and Supplementary Methods). According to Fig. 1d, 99.0 % of SNVs with MAF 0.1% or larger were expected to be captured by the present sampling strategy.

### Functional impact of very-rare variants

**Figure 2:**
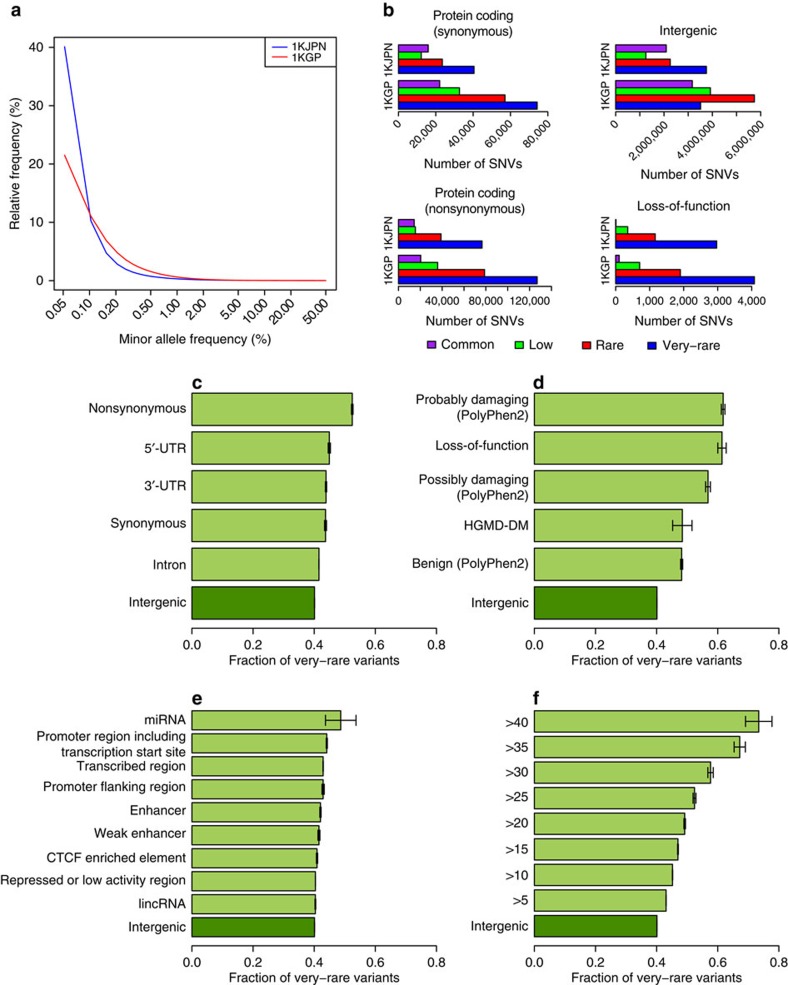
The impact of very-rare variants on genomic regions and functional categories. (**a**) The SFSs of intergenic region for SNVs of 1KJPN (blue) and 1KGP (red). (**b**) The numbers of SNVs observed in 1KJPN and 1KGP are depicted as four functional categories. The fraction of very-rare variants observed in 1KJPN are depicted with 95% binomial confidence interval according to (**c**) genomic region, (**d**) probable consequences for coding regions, (**e**) in noncoding regions and (**f**) for scaled C scores. Because the number of genotyped individuals in the confidence SNVs is different among sites because of the individual depth filter, we applied a hypergeometric projection^65^, which subsamples each variant down to a sample size of 963 (90% of 1,070 samples) to obtain the SFSs of the confidence SNVs for **a**,**c**–**f**.

The high-coverage PCR-free protocol of WGS has generated higher-power SNP discovery, especially for rare and very-rare SNVs. On comparison of the SFSs of intergenic region of 1KJPN and 1KGP SNVs, the data demonstrate a higher proportion of very-rare SNVs in the 1KJPN data set than in 1KGP phase 1 data set (Fig. 2a). In addition, although the number of SNVs in 1KGP was generally larger than that in 1KJPN, the number of very-rare variants detected in intergenic region was higher in 1KJPN than in 1KGP (Fig. 2b). These observations imply that there was less bias in discovering the very-rare variants of 1KJPN even in the intergenic region, although a possibility of faster expansion rate in 1KJPN cannot be excluded.

Because deleterious mutations are removed from populations faster than neutral mutations, SNVs observed at lower frequency in a population are indicative of purifying (negative) selection, and their selection strength differs among the various functional genomic categories. Along with this idea, the SFS has been analysed to evaluate relative influence of (mostly) negative selection on SNVs of each functional category in large sequencing projects^9^,^10^,^20^. Because we conducted the WGS without PCR amplification or exome capture, it is expected that there are less bias in variant detection between coding and noncoding regions. Therefore, the SFS of functional categories can be directly compared with intergenic region. We classified the high-confidence SNVs into predicted functional categories and evaluated the effect of purifying selection as a fraction of very-rare variants (FVRV; Fig. 2c–f). The FVRV of intergenic regions was 40.1%, which was the lowest among all categories (Fig. 2c), supporting the notion that most of the sites in intergenic regions are evolutionarily neutral. In contrast, FVRVs of noncoding regions other than intergenic were significantly higher than the FVRV of intergenic regions—introns (41.6%), synonymous (43.7%), 3′-untranslated region (UTR, 43.9%) and 5′-UTR (45.0%)—implying that a substantial fraction of noncoding regions are functional and under weak purifying selection. Similar tendencies were observed for insertions and deletions (Supplementary Fig. 4a). We conducted the same analysis with 1KGP phase I data set. In contrast to 1KJPN, the FVRVs of 5′-UTR, 3′-UTR and intron from 1KGP data set were lower than the FVRV of synonymous SNVs (Supplementary Fig. 4b). This might not be a signature of weak purifying selection on UTR and intron regions. This is rather due to the low power of SNP discovery of 1KGP in these regions where majority of them have been sequenced with low coverage in the project.

Mutations that disrupt protein and/or transcript structure are highly detrimental. This analysis reconfirmed that the FVRV of nonsynonymous transcribed SNPs (52.5%) was distinctly higher than synonymous (43.7%) and intronic (41.6%) variants (Fig. 2c). Nonetheless, the FVRV of loss of function mutations (61.4%) was much higher than nonsynonymous SNVs (Fig. 2d). In addition, we detected heterogeneity in the FVRV of nonsynonymous SNVs in terms of functional consequences predicted by PolyPhen-2, as previously reported^10^. The FVRV of SNVs that were predicted to be ‘probably damaging' was the highest (61.8%), followed by the fraction that was ‘possibly damaging' (56.8%) and finally ‘benign' (48.2%). We can also infer the impact of purifying selection on disease-causing mutations, those categorized as ‘disease mutations' in the Human Gene Mutation Database (HGMD)^31^ in terms of FVRV. The FVRV of disease mutations was 48.4%, which is very close to benign SNVs.

Although the intergenic region exhibits the lowest FVRV, the ENCODE project revealed that a large proportion of intergenic regions may be associated with biochemical activity^32^. Thus, we inferred the influence of natural selection on intergenic regions using the predicted chromatin state^33^ from the chromatin immunoprecipitation-Seq data produced by the ENCODE Consortium^32^. Among seven categories of predicted chromatin states, the SNVs observed on genomic segments bearing some functionally predicted activity exhibited higher FVRVs than repressed or low-activity regions (Fig. 2e). The difference in FVRV among chromatin states was small, but significant. This indicates weak selection on specific intergenic regions, such as promoters and enhancers, for gene regulation. Furthermore, we observed that the FVRV of microRNAs (miRNAs), but not lincRNAs, was higher, not only than for intergenic regions but also for functionally predicted ENCODE regions.

Notably, the degree of deleterious SNVs predicted by scaled C score in Combined Annotation Dependent Depletion^34^, which incorporates several annotations such as conservation metrics and regulatory information, was highly correlated with the FVRV of the 1KJPN variants (Fig. 2f). These observations clearly illustrate that this reported sequencing paradigm successfully discovered biologically relevant SNVs, most of which are rare in the Japanese population.

### Structural variants

**Figure 3:**
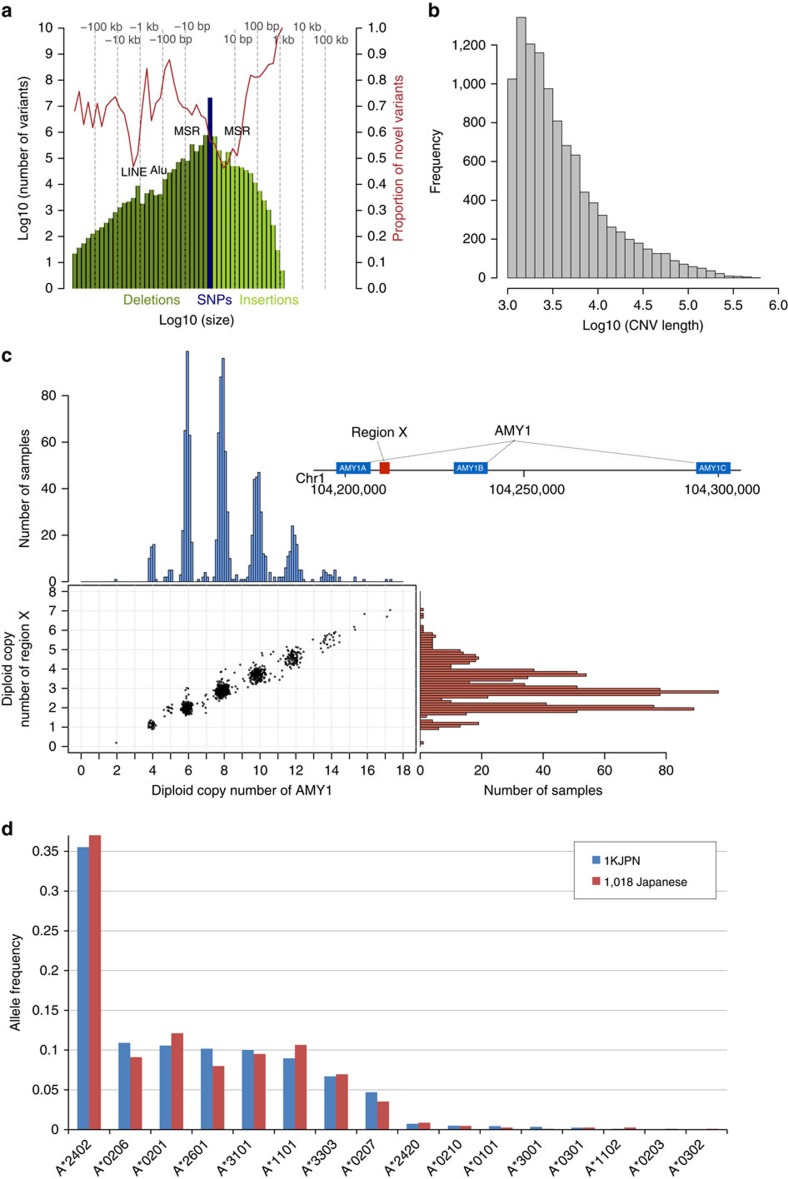
Properties of genomic variation discovered in 1KJPN. (**a**) The size-frequency spectrum of SNVs, deletions and insertions discovered by high-coverage sequencing in 1KJPN. Novelty rates are shown by the red line. Peaks corresponding to long interspersed elements (LINE), Alu and microsatellite repeat (MSR) are shown. (**b**) Size-frequency spectrum of CNVs estimated from high-coverage sequencing data in the genic regions in 1KJPN. (**c**) Histograms and scatterplot of diploid copy numbers of *AMY1* genes (blue) and region X (red) in 1KJPN. A diagram depicting the positions of *AMY1A*, Region X, *AMY1B* and *AMY1C* on chromosome 1 of GRCh37 is shown in the right top. (**d**) Allele frequencies for *HLA-A* in 1,070 individuals in 1KJPN estimated by high-coverage sequencing (blue), and 1,018 Japanese individuals typed by PCR-SSOP (red)^42^.

High-coverage sequencing data allowed us to catalogue both novel and known deletions and insertions within 1KJPN (Fig. 3a). The size-frequency spectrum showed that larger indels were less abundant than smaller ones, consistent with observations from both 1KGP^9^ and Genome of the Netherlands^20^. Notably, most of the longer insertions (>10 bp) detected in this pipeline^35^ were novel, which represents the usefulness of this high-coverage sequencing strategy (see Methods).

We also constructed a genome-wide map of CNVs overlapping with the genic region in 1KJPN, in which 25,923 CNV loci were identified; precise copy numbers were quantified on the basis of the alignment status and depth of coverage of sequence reads on individual CNV loci (Fig. 3b, Methods and Supplementary Data 1).

Among the revealed CNVs, we found that most Japanese individuals have more than two diploid copies of the salivary amylase gene (*AMY1*; Fig. 3c). The mean diploid copy number of *AMY1* within 1KJPN was 8.27, which is significantly higher than the number reported in populations who consume low amounts of starch (mean 5.44, *N*=93)^18^. This observation is consistent with the higher copy number of *AMY1* reported in high-starch populations, suggesting adaptation of an increased copy number according to dietary habits^18^. Interestingly, we found that the copy-number unit of *AMY1* was two (that is, even *AMY1* diploid copy numbers), in which the closely linked *AMY1A* and *AMY1B* loci duplicated together (Fig. 3c). Indeed, the diploid copy number of *AMY1* is proportional to the copy number of intermediate region between *AMY1A* and *AMY1B* (designated by ‘Region X' in the schematic diagram of the human reference genome in Fig. 3c), in which the relationship can be described as *y*=2*n*+2, as the diplotype model, where *y* is the diploid copy number of *AMY1*, and *n* is the diploid copy number of Region X. We also performed the digital PCR analysis to validate the estimated copy numbers in 10 samples (Methods and Supplementary Fig. 5). This means that two *AMY1* gene loci, *AMY1A* and *AMY1B*, and Region X are close to one another on a chromosome, and this region as a whole is the copy unit of the *AMY1* gene. We further confirmed that there is almost no variation in copy number outside of the *AMY1* genes. For example, diploid copy numbers of *AMY2B* and Region Y are mostly two (Supplementary Fig. 6b). This observation is consistent with the previously proposed haplotype model for the salivary amylase gene, (*AMY1A*-*AMY1B*)*n*-*AMY1C*^36^. Since the copy number of CNV loci present in a sample (gene dosage) is likely to affect the relative abundance of gene expression, this information will be useful for finding association of genetic variants with phenotypes or diseases in future follow-up studies. The CNVs reported here can be used to investigate associations with human disease phenotypes as previously reported^37^,^38^.

### HLA types

Another important aspect of deep sequencing is the ability to determine highly polymorphic gene alleles. Human leucocyte antigen (HLA) genes are highly polymorphic in the human genome, and their haplotype structures are different among human populations^39^. In this study, we have typed HLA alleles of *HLA-A*, *HLA-B* and *HLA-C* loci of 1KJPN using newly developed software called HLA-VBSeq^40^. The method is based on the alignment of sequence reads to the genomic HLA allele sequences registered in the IMGT/HLA database^41^. We successfully typed *HLA-A* alleles for most of the individuals in 1KJPN (2,063 out of 2,140 alleles) at full resolution (8-digit in HLA nomenclature). We observed that the frequencies of *HLA-A*, -*B* and -*C* alleles at 4-digit resolution (nucleotide differences that change amino-acid sequences) estimated in our study were very similar to previously published frequencies (Fig. 3d and Supplementary Fig. 6c,d), in which HLA types were determined using PCR-SSOP among a different set of 1,018 Japanese individuals^42^. Because HLA genes are crucial in determining the outcome of organ transplantation and susceptibility to infectious and autoimmune diseases^43^,^44^, this HLA typing will be particularly important for GWAS^45^ and clinical practice, such as donor–recipient matching^46^.

### Haplotyping and imputation

**Figure 4:**
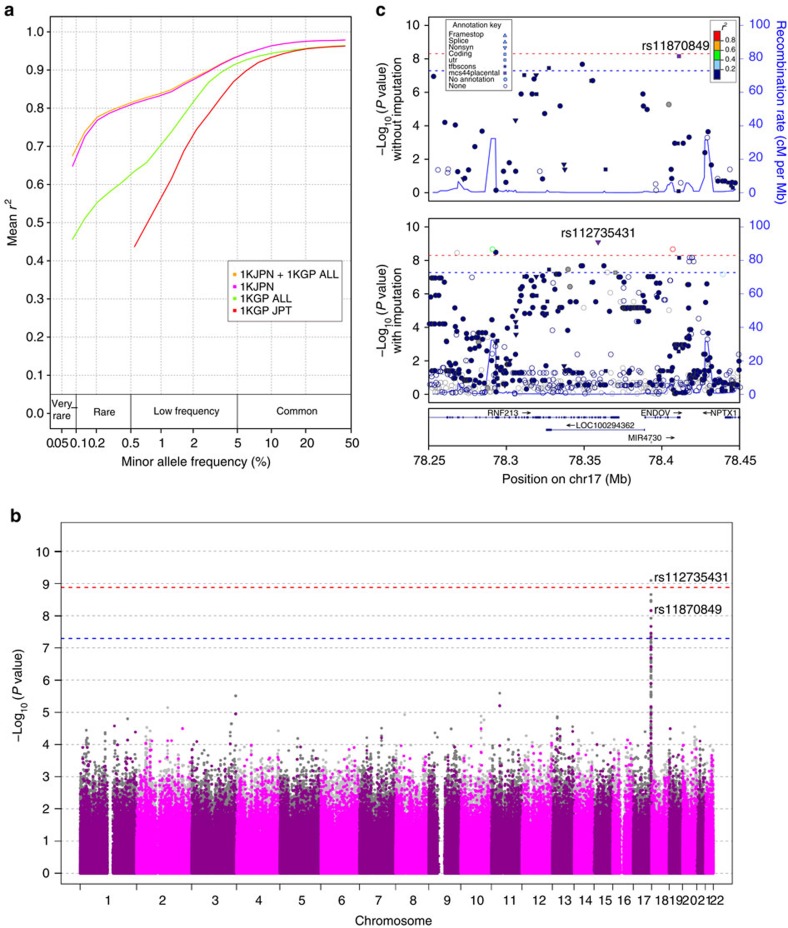
Imputation with the Japanese reference panel. (**a**) Comparison of imputation performance (*r*^2^) for four reference panels: 1,070 individuals in 1KJPN (1KJPN), 1,092 cosmopolitan samples in 1KGP (1KGP ALL), 1KJPN plus 1KGP ALL (1KJPN+1KGP ALL) and 89 Japanese individuals in 1KGP (1KGP JPT). The *x* axis represents the MAF of each panel. The *y* axis represents the averaged *r*^2^ at SNV sites that exist in both the cosmopolitan samples of 1KGP and 1KJPN. (**b**) A Manhattan plot of *P* values from GWAS of MMD. The SNV sites from the original data set and imputed markers are plotted as dots in magenta and grey, respectively. Blue and red lines display the significance threshold of the original and imputed results, respectively. Only one significant signal was identified on chromosome 17. (**c**) A plot of *P* values from GWAS of MMD with the original (non-imputed; upper panel) and imputed (lower panel) data set around the SNP exhibiting the significant signal in **b**. In the imputed result, the SNP with the highest association is a nonsynonymous variant of *RNF213*, and was reported as one of the MMD-causing variants in the original study. In contrast, from the non-imputed result the SNP with the highest association is located in the coding region of *ENDOV*.

Genome-wide genotype imputation is a statistical technique to estimate untyped genotypes from known haplotype information, which is cost-effective for GWAS with SNP arrays when compared with exome sequencing and WGS. We constructed a phased reference panel with high-sensitive SNVs plus short insertions and deletions in 1KJPN using HapMonster^47^ and ShapeIT2 (ref. 48). The performance of genotype imputation with the reference panel for 131 Japanese individuals (who were not among the individuals used to compile 1KJPN and not cryptic relatives to them; estimated identity-by-descent <0.125) was assessed by comparing their imputed genotypes and those obtained with the same sequencing protocol and variant calling pipeline that was used to generate 1KJPN. For the imputation, genotypes at sites designed in Omni2.5 were used (Supplementary Methods). We compared 1KJPN and the following three types of reference panels for genotype imputation: (i) the 1,092 cosmopolitan samples in 1KGP, (ii) the 89 JPT samples in 1KGP and (iii) the 1KJPN plus 1KGP. From the comparison, the highest *r*^2^ value (the measure of imputation accuracy) was achieved with 1KJPN plus 1KGP, especially in variants with MAF≤5%; the mean *r*^2^ values were 0.47 for very-rare SNVs, 0.66 for rare SNVs and 0.78 for low frequency SNVs (Fig. 4a). The significant improvement of imputation accuracy using the 1KJPN data suggests the importance of construction and examination of a population-specific reference panel.

To illustrate the effectiveness of the genotype imputation strategy in GWAS, we performed imputation from genotyped data set in a case–control study of MMD^49^ with 1KJPN (Supplementary Methods). MMD is a progressive cerebrovascular disorder caused by blocked arteries at the base of the brain and has unusually high prevalence among the Japanese. The data set contains the genotypes with Illumina HumanOmni1-Quad BeadChip for 72 Japanese MMD patients and 45 healthy controls from HapMap JPT collection. We performed GWAS for the imputed data sets with the same statistical parameters used in the original study^49^. A synonymous SNP rs11870849 located at the coding region of *ENDOV* (chr17:78,411,073) with a *P* value of 6.95 × 10^−9^ (*χ*^2^-test) was identified as the highest association SNP (Fig. 4c top) from the original (non-imputed) data set (Fig. 4b). In contrast, by the imputation employing 1KJPN (Fig. 4c bottom), a nonsynonymous SNP rs112735431 located in *RNF213* (chr17:78,358,945; MAF=0.0089 in 1KJPN) was identified as the highest association SNP with a *P* value of 8.07 × 10^−10^ over a significance threshold of a *P* value <5.06 × 10^−9^. This SNP was confirmed by fine mapping of chromosome 17q ter locus with another set of SNPs followed by targeted sequencing^49^, which was suggested to be a major MMD causal variant^49^,^50^. Thus, we have directly inferred the causal SNP with our 1KJPN reference panel.

### Functional variant load in individuals

We summarized the number of disease-causing variants in autosomes per individual for each derived allele frequency (Table 2 and Supplementary Methods). On average, one individual has 11.2 disease-causing alleles in terms of disease-causing mutation (DM) category of HGMD^31^ (9.6 as heterozygous and 1.6 as homozygous in the high-confidence SNVs). Similarly, we calculated the number of nonsense (stop-gained) variants per individual on the high-confidence SNVs. Each individual has on average 41.6 heterozygous and 12.1 homozygous stop-gained SNVs. These estimates are reasonably consistent with those in East Asian populations in 1KGP (JPT, CHB and CHS). Although there are reports on higher mutational loads in founding populations^13^,^21^,^51^, our analysis of mutational load did not show clear difference between Japanese and other East Asian populations. In terms of nonsense mutation, the estimate of individual load in 1KJPN is consistent with those in the analysis of the Dutch population^20^. In 1KJPN, 3,505 SNVs were annotated as stop-gained, while among them only 4.5% were annotated as phenotypic variants in HGMD. This low proportion suggests that the biological or pathological effects of most stop-gained SNVs are yet to be revealed.

Incidence rates for inherited diseases vary among populations^52^. For example, the incidence of inherited metabolic diseases was found to be 1 in 9,330 in Japan, while among those of European ancestry, metabolic diseases were more prevalent (1 in 4,000–5,000)^53^. Information of risk allele frequencies among populations will be useful for estimating penetrance and identifying additional causes of disease, such as other pathogenic loci or environmental factors. Indeed, we found significant differences of allele frequency for HGMD variants between 1KJPN and each of 14 populations in 1KGP. The 2,638 SNVs in HGMD were significantly different (*P* value<10^−5^; Fisher's exact test) in at least one population of the 1KGP. Among them, 36 SNVs were annotated as disease-causing mutations. For example, SNP rs1047781 at chromosome 19:49,206,631 (causing an Ile129Phe change) in the *FUT2* gene (encoding fucosyltransferase 2) showed a drastic difference in the non-reference allele frequency between the 1KJPN (0.38) and 1KGP CEU (0.00). The *FUT2* gene encodes an enzyme for secreting ABH antigens, and the locus is known as a classic human secretor locus^54^. The frequency of homozygotes of this variant was 0.141 in 1KJPN, and this is consistent with the fact that ∼15% of Japanese individuals are *FUT2* nonsecretors^55^. Recent studies showed that this SNP rs1047781 was associated with the levels of tumour biomarker and vitamin B12 (refs 56, 57).

## Discussion

From the high-coverage WGS data collected in the ToMMo cohort project, we have constructed 1KJPN, a reference panel of 1,070 Japanese individuals. This panel includes a highly accurate and comprehensive collection of 21.5 million SNVs ranging from very-rare (observed MAF≤0.1%) to common (>5%) variants. Considering the demographic history of 1KJPN inferred from the SFS of intergenic region, the data cover almost all SNVs (99.0%) that exist in the Japanese population having a minor frequency of 0.1% or greater (Fig. 1d). Furthermore, we would find rarer variants if additional samples will be sequenced. For instance, while we have detected 60.5 % of SNVs with MAF>0.01% in this study, it is expected that the detection rate will rise to 81.6 % and 96.2 % for the sample size 3,000 and 8,000, respectively (Fig. 1d). Since most of very-rare variants likely arose within 5,000–10,000 years^10^, they are essentially population-specific. Hence, most of them would be difficult to be imputed from a reference panel that was constructed from diverse genetic backgrounds. Indeed, we showed here significantly improved imputation accuracy from 1KJPN data when compared with 1KGP (Fig. 4 and Supplementary Fig. 7).

In 1KJPN, we found the signature of purifying selection on SNVs in regulatory elements located in both coding and noncoding sequences. This observation illustrates that this SNV set is expected to contain many very-rare variants that can be associated with diseases, and thus should be useful in future GWASs to fully capture causal variants.

This high-coverage strategy has also defined fine characteristics of SVs, CNVs and HLA types in a population scale. Although a substantial number of novel SVs was discovered by the high-coverage sequencing, we still had difficulty in discovering insertions longer than 100 bp because of the limitation of sequence read lengths (162 bp paired-end). In addition, simple repeat regions and structurally complex regions (for example, centromeric and telomeric sequences) were not covered in our SV discovery. To build a more comprehensive reference panel with SVs, alternative approaches, such as the use of longer sequence reads, would be necessary.

Since the cost of WGS is gradually decreasing, population-wide sequencing, as in the present study, will become a reasonable approach to discover the full spectrum of SNVs, SVs and CNVs in a reliable manner. However, it remains challenging to sequence more than tens of thousands of samples in cohort projects even with the sophistication of current sequencing technologies. Thus, a hybrid strategy that combines both sequencing and genotyping with a customized SNP array optimized to a specific population background, for example, Japonica Array^58^, becomes highly desirable. Our hope is that 1KJPN will foster basic research by amalgamating more accurate genotype imputation in cohort studies with medical information, and thereby aid in constructing an advanced medical system to improve the quality of health-care services.

## Methods

The Supplementary Note in this manuscript lists details of samples, data generation, selection protocols with the SNP array and also informs the bioinformatics not covered in this section, for example, the estimation of HLA types, imputation and simulation of population genetics.

### Sample information

This project was performed as part of the prospective cohort study at the ToMMo, with the approval of the ethical committee of the Tohoku University School of Medicine. The samples used here were from the cohort participants, all of whom gave their written consent. In our informed consent to the participants to our cohort project, whole-genome data including ‘sequenced data, variant calls and inferred genotypes' are securely controlled under the Materials and Information Distribution Review Committee of Tohoku Medical Megabank Project and the data sharing to researchers are discussed in each research proposal by the review committee.

The current status of our committee is to put the part of the SNP frequency data as open data in the National Bioscience Database Center website (http://humandbs.biosciencedbc.jp/en/). Every year, the data availability policy will be updated with the review committee.

### Whole-genome sequencing

To prevent sample mix-up, samples were handled in 96-well plates during the course of the library construction. The genomic DNA samples were diluted to a concentration of 10 ng μl^−1^ using a laboratory automation system (Biomek NXP, Beckman), and fragmented using a 96-well plate using the DNA sonication system (Covaris LE220, Covaris) to a target size of 550 bp, on average. The sheared DNA was subjected to library construction with the TruSeq DNA PCR-Free HT sample prep kit (Illumina) using a Bravo liquid-handling instrument (Agilent Technologies). Finally, the completed libraries were transferred into 1.5 ml tubes that were labelled with a barcode, and were then denatured and neutralized, and processed for library QC.

The library quantitation and QC were performed with quantitative MiSeq (qMiSeq), a newly developed method for library quantitation. In this protocol, 8 or 10 μl of prepared libraries was denatured with an equal volume of 0.1 N NaOH for 5 min at room temperature, and diluted with a 49-fold volume of ice-cold Illumina HT1 buffer. Overall, 50 μl of 96 denatured libraries including three control samples (those examined earlier on HiSeq) were pooled. Then, 60 μl of the pooled library was diluted with 540 μl of ice-cold Illumina HT1 buffer and analysed with MiSeq using a 25-bp, paired-end protocol. We utilized the index ratio determined by the MiSeq sequencer, as a relative concentration to determine the run condition for the HiSeq run. To share the details of qMiSeq, we have published a methodology paper^27^. In addition to qMiSeq, an electrophoretic analysis using the Fragment Analyzer (Advanced Analytical) software was performed as a part of the library QC.

DNA libraries were analysed using the HiSeq 2500-sequencing system, according to the manufacturer's protocol. A TruSeq Rapid PE Cluster Kit (Illumina), and one-and-a-half TruSeq Rapid SBS Kit (200 cycles, Illumina), were used to perform a 162-bp, paired-end read in Rapid-Run Mode. On the basis of the qMiSeq results, the libraries had been diluted to the appropriate concentrations and were used for on-board cluster generation (Illumina). We routinely checked the cluster density at the first base report and decided whether to continue the analysis depending on the density (∼550–650 K mm^−2^).

### SNV step 1 alignment and variant call for each sample

The sequence reads from each sample were aligned to the reference human genome (GRCh37/hg19) with the decoy sequence (hs37d5). Two aligners, Bowtie2 (version 2.1.0) with the ‘-X 2000' option, and BWA-MEM (ver. 0.7.5a-r405) with the default option, were used. To call the SNVs, the Bcftools software (ver. 0.1.17-dev) and the Genome Analysis Toolkit (GATK version 2.5–2) were, respectively, applied to each aligned result (Step 1 in Supplementary Fig. 2). For each sample, the read depth of each SNV position was calculated for the downstream filtering steps (Supplementary Fig. 2; middle-bottom in Step 1). Here the read depth represents the total number of sequence reads aligned to the SNV position, with the mapping quality being more than or equal to 5.

We compared the SNV calls and the genotyping results from the HumanOmni2.5–8 BeadChip for the same samples to evaluate the precision and the power (=recall) at designed positions in the SNP array. The precision, as calculated using Bowtie2 with the Bcftools variant caller (Bowtie2+Bcftools), was always better than that obtained using BWA-MEM with GATK. On the contrary, the power obtained using BWA-MEM+GATK was always better than that obtained with Bowtie2+Bcftools (Supplementary Data 2). These results are consistent with a previously conducted analysis^59^. Thus, the SNVs obtained using BWA−MEM+GATK were considered the result of highly sensitive SNV detection (trying to find as many SNVs as possible), whereas the SNVs that were obtained using bowtie2+Bcftools were used as the candidate of the high-confidence SNVs to apply to the downstream filtering steps. Hereafter, the term ‘SNVs' will refer to variants called using Bowtie2+bcftools.

### SNV step 2 genotype depth filter for each individual

We filtered out the genotype calls that had an extraordinarily low or high read depth. For each sample, we determined the range of the read depth on the SNVs to be retained, according to the precision and the recall from the comparison with the genotyping results of the HumanOmni2.5–8 BeadChip. The SNVs were grouped by read depth of next generation sequencing (NGS) (we only used the sequenced reads with a mapping quality more than or equal to 5, to avoid the reads being aligned to multiple chromosomal locations), and then precision and recall were calculated for each group (Fig. 1a and Supplementary Fig. 2; the middle plot in Step 2). The SNVs in the groups with a precision value >0.998 were extracted as reliable SNVs (Supplementary Fig. 2 SNV Filter; the left plot and figure in Step 2). In this step, 2.0% of the segregating sites were filtered out (Step 2 in Supplementary Table 1).

### SNV step 3 SNV depth filter for each locus

We then filtered out the SNV loci in which more than 10 % of the samples were not genotyped because of the depth filter applied in the previous step. For example, the SNV calls in such loci might suffer from an extraordinarily high or low depth, because of the read alignment to repetitive sequences. The pass ratio of the SNV sites was calculated (Supplementary Fig. 2; the middle figure in Step 3). In this step, 4.99% of the SNV sites were filtered out (Step 3 in Supplementary Table 1).

### SNV step 4 genome complexity filter

It is difficult to call SNVs in low-complexity genomic regions, such as short tandem repeats. The RepeatMasker programme annotated 56% of genomic regions as low-complexity regions. The precision of each annotated group was calculated and compared with the genotyped SNP array of the same sample (Supplementary Fig. 2; the left table in Step 4). The precision of the SNV calls was calculated for each genomic region by comparing them with the genotype calls obtained with the HumanOmni2.5–8 BeadChip. Then, the SNVs in the genomic regions whose precision was ≤0.997 were removed (Supplementary Fig. 2; the right figure in Step 4), that is, Alu, ERVK, Low_complexity, Satellite, Simple_repeat, TcMar-Mariner and others (more detail in Supplementary Data 3). In this step, 14.22% of SNVs were filtered out (Step 4 in Supplementary Table 1).

### SNV step 5 tool bias filter

To control the bias of the tool used in Step 1, we only retained the SNV sites that were also discovered using the other method. Thus, we removed the SNVs that were not detected by BWA-MEM+GATK, that is, the highly sensitive SNVs. At the top of the figure in Step 5 (Supplementary Fig. 2), the leftmost variant was not detected using the other tool, and thus those SNVs were not used in the downstream analysis. In this step, 0.57% of the SNVs were filtered out (Step 5 in Supplementary Table 1).

### SNV step 6 population genetics filter

Finally, we filtered out the SNVs with a HWE test *P* value<10^−5^, using the genotype frequencies of the SNVs determined in Step 5. This filter removed the SNV-genotype frequencies that are not consistent with the HWE. Most of the SNVs that were filtered out might be artefacts from the incompleteness of the reference genome, or from systematic alignment errors. In this step, 1.03% of the SNVs were filtered out, and in the end, 77.20% of the raw SNVs were mined through these filtering steps (Step 6 in Supplementary Table 1).

### SNV validation

The autosomal SNVs with a MAF≤0.5%, a MAF of 0.5–1%, a MAF of 1–5% and a MAF >5% were subjected to validation. For each frequency, the MassARRAY design (Sequenom) was applied to 1,000 potential variants called in at least one of the 12 representative samples, which were randomly selected from 1,070 samples (in the MassArray experiments, as maximum 36 SNVs can be evaluated at once. There are some limitations for the combination of SNVs. Thus, we have started from enough 1,000 SNV candidates in the SNV validation). Among the SNPs that passed an assay design step (809, 813, 832 and 819 sites for each frequency, respectively), 322 sites (108, 70, 72 and 72 sites for each frequency, respectively) were selected and analysed using the Sequenom MassARRAY (Sequenom). The same sites were also analysed using amplicon-sequencing.

The MassARRAY analyses were performed according to the manufacturer's protocol. The design of the PCR and the extension primers was performed using the Assay Design suite (Sequenom). The genomic regions containing potential variants were amplified by multiplex PCR (35 or 36 sites for each region). The single-base extension reaction was followed by alkaline phosphatase treatment, and reaction mixtures were purified with resin and spotted on 96-well SpectroCHIPs using a Nanodispenser. SpectroCHIPs were analysed using the MassARRAY system. Variant calls were performed using the SpectroTyper software (ver. 4.0). The Sequenom validation was applied to 94 samples (including the 12 validation samples). If a variant was confirmed in at least one of the twelve validation samples, the variant was marked as being true. In the event that the call rate was less than 80%, or that a nonspecific, single-base extension was observed in the no-template control, the assay was considered unreliable and was marked as a no-call.

The genomic regions examined using the MassARRAY were amplified using a two-step PCR to add Illumina sequencing adaptors. In total, 322 regions of the 12 samples were sequenced with 76 bp, paired-end reads using a MiSeq sequencer with the MiSeq Reagent Kit v3. Index sequences (Illumina D701-D712) were used to distinguish the 12 validation samples. To validate the SNVs, we performed the following data analysis. (1) For each SNV site, the nucleotide sequences comprising both the 300 bp upstream and downstream of the reference allele, and of the alternative allele, were prepared, (2) the sequence reads were aligned to these reference sequences using BWA-MEM^60^ and (3) based on the depth of coverage at each site, the SNV call was validated. The regions of low coverage (less than 30 × ) were marked as a no-call. If a variant was confirmed in at least one of the 12 validation samples, it was marked as being true.

Sanger sequencing was performed using the BigDye terminator cycle sequencing kit, v.3.1 (Applied Biosystems), in accordance with the manufacturer's instructions. The SNVs that had discordant genotype calls between the HiSeq and the MassARRAY analyses, or between the HiSeq and the amplicon-sequencing analyses, were further validated using Sanger sequencing. The genomic DNA fragments containing one of the SNVs requiring validation were amplified by PCR, and these fragments were then sequenced in both the 5′ and the 3′ directions. The oligonucleotide sequences used for PCR and for sequencing are available on request.

From the validated 322 sites, 282 sites were remained in the high-confidence SNVs set. Finally, 62, 124 and 96 SNVs were the validation targets in rare and very-rare, low and common MAF class, respectively. If either the MassARRAY or the amplicon-sequencing analysis turned out to yield a no-call, the SNV site was also treated as a no-call. The SNV sites that were validated by both methods were treated as true positives. If the two methods produced inconsistent genotypes, we assumed that the genotypes determined by Sanger sequencing are true. The FDR was calculated by dividing the number of false variants by the sum of the true and false variants (Supplementary Table 2).

To validate the newly discovered SNVs in this study, we also conducted the validation experiment using a custom Illumina BeadChip array. The custom array contained probes of 25,317 novel SNVs that were arbitrarily picked from the novel variants in the high-confidence SNV set. These SNVs include SNVs in intergenic regions as well as in coding regions, some of which were of biological interest (for example, nonsynonymous or stop-gained variants).

The genotyping of 854 samples, which are part of the 1KJPN, was conducted using this custom array with the same protocol used for the HumanOmni2.5–8 BeadChip genotyping described above. We validated the SNV discovery at the designed sites, and calculated per site FDR of novel SNVs. If a SNV is discovered in several samples and more than one samples could detect the same SNV among these samples by using the SNP array, then the SNV is considered to be correctly discovered and the SNV is counted as true. Otherwise, the SNV is counted as false. Finally, the total FDR for each category (common, low, rare and very-rare) in Supplementary Table 3 was calculated as follows: overall per site FDR=(FDR of known SNV) × *f*_known_+(FDR of novel SNV) × (1−*f*_known_), where *f*_known_ is the fraction of SNVs listed in the single nucleotide polymorphism database (dbSNP) and FDR of known SNV as 0.

### Indel validation

Autosomal indels ranging in size from 1 to 30 bp were subjected to validation. The 150 potential indels called in at least one of the twelve representative samples, which were randomly selected from the total 1KJPN samples, were applied to the MassARRAY design (Sequenom). Among the 98 indels that passed the assay design step, 95 sites were randomly selected and analysed using amplicon sequencing.

The 95 regions of the 12 samples were sequenced with 76-bp, paired-end reads using a MiSeq sequencer with the MiSeq Reagent Kit, v3. Index sequences (Illumina D701-D712) were used to distinguish between the 12 validation samples. To validate the indel calls, we performed the following data analysis: (i) for each indel site, the nucleotide sequences comprising both the 300 bp upstream and downstream of the reference allele, and of the alternative allele (either insertions or deletions), were prepared, (ii) the sequence reads were aligned to these reference sequences using BWA-MEM and (iii) based on the depth of coverage at each site, the indel call was validated. The regions of low coverage (less than 100 × ) were marked as no-calls. If an indel was confirmed in at least one of the twelve validation samples, it was marked as being true.

If the amplicon-sequencing analysis turned out to yield a no-call, we tried to determine the genotypes using Sanger sequencing. If Sanger sequencing could not determine the genotypes, the variants were treated as a no-calls. The FDR was calculated by dividing the number of false variants by the sum of the true and false variants (Supplementary Table 2).

### Variant annotation

The effects of the coding and the intergenic variants were predicted by the SnpEff software (ver. 3.3c), based on the gene annotation model of GENCODE version 17. The functional categories of synonymous, nonsynonymous, intron, 5′-UTR and 3′-UTR were applied to the SNVs whose transcript label was ‘protein-coding'. For the insertion and the deletion variants, the variants on the protein-coding region were labelled according to the impact record of the SnpEff annotation. The category of ‘intergenic' was applied for intergenic regions when no annotation was assigned to the transcript label. The functional consequences of nonsynonymous SNVs were retrieved from the PolyPhen-2 records of the dbNSFP 2.0 database^61^. The scaled Combined Annotation Dependent Depletion scores (C scores)^34^ were added to each SNV by intersecting the list of the precomputed C scores with all possible SNVs. The predictions of the chromatin state were obtained from the ENCODE Project. The predictions were categorized into seven chromatin states—TSS: predicted promoter region including TSS; PF: promoter-flanking region; E: enhancer; WE: weak enhancer or open chromatin *cis*-regulatory element; CTCF: CTCF-enriched element; T: transcribed region; and R: repressed or low-activity region. The combined predictions of ChromHMM^33^ and Segway segmentation^62^ in the GM12878 cell line were assigned to the respective SNVs. The RNA regions (miRNA, lincRNA (long intergenic non-coding RNA), small nuclear RNA (snRNA) and snoRNA (small nucleolar RNA)) of the SNVs were annotated from several sources. The annotations of miRNA and lincRNA were performed according to the miRBase^63^ and the Human lincRNA Catalog^64^, respectively. Only the mature parts of the miRNAs were used for annotation (Supplementary Fig. 4). The annotation model of GENCODE17 was used for annotation of the snRNAs and of the snoRNAs.

### Site frequency spectrum

The SFSs were analysed to assess the impact of natural selection on each category of SNVs. The number of genotyped individuals to obtain the high-confidence SNVs varies from one SNV to another because of the individual depth filter (Step 2 in Supplementary Fig. 2). Thus, we applied the hypergeometric projection^65^, in which each variant was downsampled to a uniform sample size across all SNV sites. The downsampled size was 963, which was the minimum number of genotyped individuals to obtain the high-confidence SNVs. To directly compare the SFSs in the 1KGP with those in the 1KJPN, we also downsampled the SNVs of the 1KGP to the uniform sample size of 963. The FVRV was calculated by dividing the number of very-rare SNVs by the total number of SNVs within the resampled SFS.

### Structural variants

SVs were called using the BreakDancer^66^ software (ver. 1.1), the Pindel^67^ software (ver. 0.2.5a3) and the Haplotype Caller software implemented in the Genome Analysis Toolkit^68^ (version 2.5–2), from the binary of sequence alignment/map (BAM) files constructed with BWA-MEM (version 0.7.5a-r405). For the insertions and deletions of a length <100 bp, calls from Haplotype Caller were used. For the deletions of a length between 100 bp and 1 M bp, calls from Pindel (≥100 bp) and BreakDancer (≥1 k bp) were used, and merged into one unified call set, as described in the integrated structural variation pipeline^35^. SV calls that were overlapping by ≥80% were merged into one unified call. The start and end positions of the merged call were defined as the mean of the start and the end positions of the original calls, respectively. The novelty rates of SV calls were calculated based on the dbSNP 138 database and the Database of Genomic Variants, as of 23 July 2013. The insertion calls whose nearest known insertion was within 10 bp, and the deletion calls that overlapped by ≥50% with a known deletion, were regarded as known calls.

### Whole-genome identification of genic CNVs

CNVs were called with the CNVnator^69^ software (ver. 0.3), using default options and a 100-bp bin size. We filtered out calls when 90% of the bases of the called region were ‘N' in the hg19 reference genome, or if ‘cnvnatorP1' or ‘cnvnatorP2' was ≥1. The CNV calls that overlapped by ≥80% were merged into one unified call. The start and the end positions of the merged calls were defined as the mean of the start and the end positions of the original calls, respectively. The autosomal CNVs whose biallelic MAF was ≥1%, length was ≥1,000 bp and positions were overlapping with a human gene annotation in Ensembl (release-75) were analysed. The copy numbers of each CNV call in the 1KJPN that overlapped with human gene loci are summarized in Supplementary Data 3.

### Diploid copy-number estimation and validation of the *AMY1* genes

First, we counted the number of reads that aligned to the following regions: *AMY2B* (chr1:104096436–104122156), *AMY1A* (chr1:104,096,436–104,207,173), *AMY1B* (chr1:104,230,036–104,239,302), *AMY1C* (chr1:104,293,027–104,301,312), Region X (chr1:104,210,000–104,211,500), Region Y (chr1:104,350,000–104,400,000) and Region Z (chr1:104,400,000–104,450,000), where the genomic coordinates (hg19 reference genome) are illustrated (see Fig. 3c and Supplementary Fig. 6a,b). Region Z was chosen on the basis of the mappability of the reference genome so that the depth of coverage in each region across the whole genome in each sample can be determined on the basis of the average depth of this region. We then normalized read counts in these regions, by dividing them by half of the counts in Region Z, so that the normalized counts became two in diploid regions. The sum of the normalized counts in *AMY1A*, *AMY1B* and *AMY1C*, and the normalized counts in Region X, were calculated for each sample (Fig. 3c). Region X is located between the *AMY1A* and *AMY1B* regions, and most of the reads were uniquely aligned to this region. Three *AMY1* gene loci (*AMY1A*, *AMY1B* and *AMY1C*) in the reference genome are very similar to each other (having more than 99.9% sequence identity), and hence it is difficult to distinguish one locus from another. The normalized coverages in *AMY1* and in Region X were highly correlated (Fig. 3c), which suggests that the locus that contains *AMY1A* and *AMY1B* is a copy unit of the salivary amylase gene. While all samples had two diploid copies of Region Y, 17 samples had more than two diploid copies of *AMY2B*.

Digital PCR was performed with the QuantStudio 3D Digital PCR System (Life Technologies) according to the manufacturer's protocol. Diploid copy numbers of the *AMY1* gene in 10 samples (whose diploid copy numbers were predicted to be six, seven or eight) were determined using predesigned TaqMan Copy Number Assay (Hs07226362_cn for *AMY1A*, *AMY1B* and *AMY1C*, Life Technologies) and normalizing with TaqMan Copy Number Reference Assay RNase P (Life Technologies). The PCR reaction mixture contained 12 ng of MspI-digested genomic DNA, 7.5 μl of QS3D digital PCR Master Mix (Life Technologies), 0.75 μl of TaqMan Copy Number Assay and 0.75 μl of TaqMan Copy Number Reference Assay in a final volume of 15 μl. The data were analysed with QuantStudio 3D AnalysisSuite (Life Technologies), and the quantification of the target genomic region was presented as the number of copies per microlitre of PCR mixture. The estimated diploid copy numbers of *AMY1* with WGS were consistent with the result obtained with the digital PCR analysis (Supplementary Fig. 5).

## Additional information

**Accession code:** Sequence data, variant calls and inferred genotypes will be available on request after approval of the Ethical Committee and the Materials and Information Distribution Review Committee of Tohoku Medical Megabank Project. Part of the data is available as open data from the National Bioscience Database Center website (http://humandbs.biosciencedbc.jp/en/) under the accession ID hum0015.

**How to cite this article:** Nagasaki, M. *et al.* Rare variant discovery by deep whole-genome sequencing of 1,070 Japanese individuals. *Nat. Commun.* 6:8018 doi: 10.1038/ncomms9018 (2015).

## Supplementary Material

Supplementary Information Supplementary Figures 1-7, Supplementary Tables 1-3, Supplementary Methods and Supplementary References

Supplementary Data 1 Whole-genome identification of genic CNVs

Supplementary Data 2 Precision and recall of SNVs

Supplementary Data 3 Precision and recall of SNVs of the RepeatMasker annotation

## Figures and Tables

**Table 1:** Summary of WGS of Japanese individuals and variant detection in autosomes.

*Info*		
Total samples		1,070
Total raw bases		100.4 trillion bases
Mean sequenced depth		32.4 ×
		
		
*SNVs*	High-sensitive SNVs	High-confidence SNVs
Total	29,588,649	21,221,195
Number of known variants*	12,308,520	9,219,783
Number of novel variants*	17,280,129	12,001,412
Novelty rate	58.40%	56.55%
Average number per sample	3,886,081	2,716,853
Average individual heterozygosity	2,252,841	1,532,773
		
	*Length*	
*Deletions*	1 bp≤length<100 bp	100 bp≤length
Number of sites overall	1,969,302	47,343
Number of novel variants†	1,429,636	—
Novelty rate	72.60%	—
Number of inframe/frameshift	3,112/4,454	—
Average number per sample	190,857	2,654
		
		
	*Length*	
*Insertions*	1 bp≤length<100 bp	100 bp≤length
Number of sites overall	1,384,230	9,354
Number of novel variants†	1,037,839	9,354
Novelty rate	74.98%	—
Number of inframe/frameshift	1,577/2,506	—
Average number per sample	159,359	45

SNV, single-nucleotide variant; WGS, whole-genome sequencing.

All data listed here are limited to the autosomal genome.

^*^Comparison based on dbSNP build 138.

^†^The decision of novel sites is described in Methods.

**Table 2:** Individual variant load in coding regions.

**Variant type**	**Population**	**Very-rare (<0.1%)**		**Rare (0.1–0.5%)**		**Low (0.5–5%)**		**Common (>5%)**		**Total**	
		**Mean**	**s.d.**	**Mean**	**s.d.**	**Mean**	**s.d.**	**Mean**	**s.d.**	**Mean**	**s.d.**
*Heterozygous*											
HGMD-DM*	1KJPN (1,070) high confidence	0.640	0.814	1.039	1.016	4.757	2.246	3.179	1.604	9.619	3.032
	1KJPN (1,070) high-sensitive	0.675	0.848	1.107	1.051	4.905	2.261	4.388	1.787	11.074	3.136
	1KGP JPT (89)	NA	NA	NA	NA	6.270	2.503	4.169	1.829	10.438	2.969
	1KGP CHB (97)	NA	NA	NA	NA	5.536	2.381	4.464	1.921	10.000	3.218
	1KGP CHS (100)	NA	NA	1.470	1.359	4.320	2.049	3.680	1.803	9.470	2.798
											
Stop-gained†	1KJPN (1,070) high-confidence	2.385	1.550	1.563	1.294	8.679	2.486	29.017	4.327	41.644	5.358
	1KJPN (1,070) high-sensitive	2.624	1.616	1.777	1.376	6.008	2.402	42.125	4.878	52.535	5.795
	1KGP JPT (89)	NA	NA	NA	NA	8.685	2.987	39.337	5.261	48.022	6.166
	1KGP CHB (97)	NA	NA	NA	NA	9.742	3.215	37.845	5.593	47.588	6.777
	1KGP CHS (100)	NA	NA	3.860	2.433	6.580	3.085	36.070	4.860	46.510	5.947
											
*Homozygous*											
HGMD-DM*	1KJPN (1070) high-confidence	0.001	0.031	0.003	0.053	0.048	0.230	1.570	1.126	1.621	1.145
	1KJPN (1,070) high-sensitive	0.000	0.000	0.003	0.053	0.050	0.234	1.862	1.235	1.914	1.251
	1KGP JPT (89)	NA	NA	NA	NA	0.022	0.149	1.899	1.244	1.921	1.227
	1KGP CHB (97)	NA	NA	NA	NA	0.052	0.222	2.021	0.989	2.072	1.003
	1KGP CHS (100)	NA	NA	0.000	0.000	0.000	0.000	0.000	0.000	2.110	1.118
											
Stop-gained†	1KJPN (1,070) high-confidence	0.005	0.081	0.004	0.061	0.753	0.747	11.303	2.713	12.064	2.813
	1KJPN (1,070) high-sensitive	0.008	0.101	0.008	0.101	0.099	0.302	12.101	2.853	12.217	2.851
	1KGP JPT (89)	NA	NA	NA	NA	0.067	0.252	11.315	2.898	11.382	2.914
	1KGP CHB (97)	NA	NA	NA	NA	0.052	0.222	12.093	2.758	12.144	2.769
	1KGP CHS (100)	NA	NA	0.000	0.000	0.070	0.326	12.900	3.047	12.970	3.096

CHB, Han Chinese in Beijing, China; CHS , Han Chinese South, China; HGMD, Human Gene Mutation Database; JPT, Japanese in Tokyo, Japan; 1KGP, 1000 Genomes Project; 1KJPN, reference panel of 1,070 Japanese individual; NA, not available; ORF, open reading frame; SNV, single-nucleotide variant.

SNV sites with reliable ancestral states were used.

^*^HGMD-DM (disease-causing) alleles were analysed if they are derived alleles and alternative (non-reference) alleles.

^†^We selected stop-gained alleles if they are derived alleles and alternative (non-reference) alleles. We discarded stop-gained SNVs if the proportion of truncated ORF is less than 5%.
