# Supplementary material for: Rare variant discovery by deep whole-genome sequencing of 1,070 Japanese individuals
Source: Nat Commun. 2015 Aug 21;6:8018. doi: 10.1038/ncomms9018 (PMC4560751; doi:10.1038/ncomms9018)
Supplement: Supplementary Information — Supplementary Figures 1-7, Supplementary Tables 1-3, Supplementary Methods and Supplementary References [file ncomms9018-s1.pdf]

a

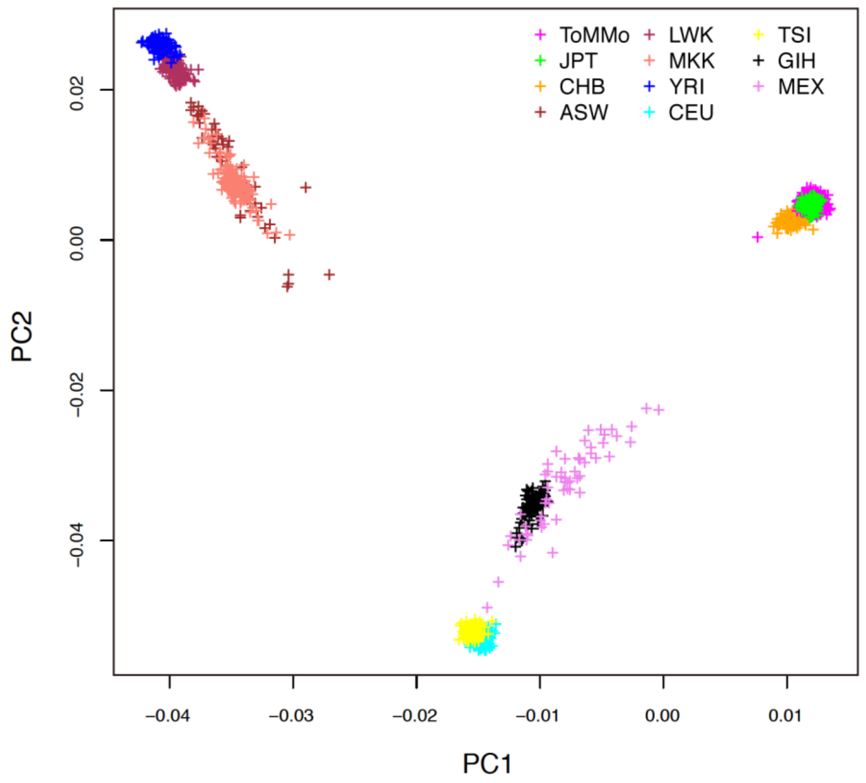
ToMMo
JPT
CHB
ASW
LWK
MKK
YRI
CEU
TSI
GIH
MEX
PC2
PC1
0.02
0.00
−0.02
−0.04
−0.04
−0.03
−0.02
−0.01
0.00
0.01

b

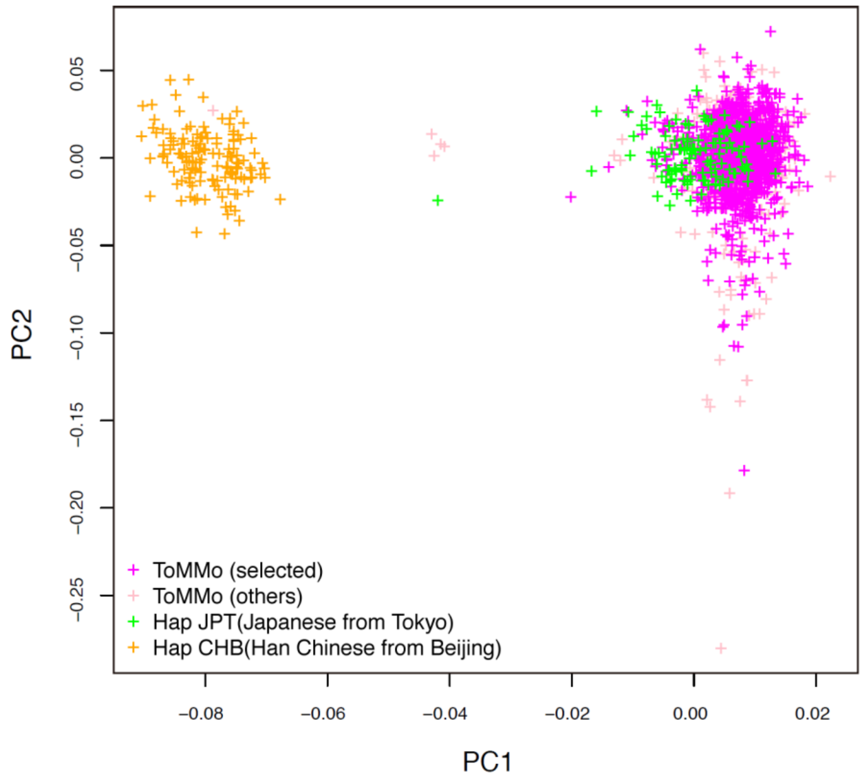
ToMMo (selected)
ToMMo (others)
Hap JPT(Japanese from Tokyo)
Hap CHB(Han Chinese from Beijing)
PC2
PC1
0.05
0.00
−0.05
−0.10
−0.15
−0.20
−0.25
−0.08
−0.06
−0.04
−0.02
0.00
0.02

Supplementary Figure 1. Population structure of ToMMo samples.

Principal component analysis (PCA) was conducted with the smartpca program in EIGENSOFT. The individuals were plotted in two-dimensional graphs, with the first (x-axis) and the second (y-axis) components. (a) PCA plot of 1,553 ToMMo individuals, along with individuals from the HapMap project. (b) The PCA plot of ToMMo individuals with the HapMap East Asian individuals (JPT and CHB). The selected 1,070 ToMMo individuals for the reference panel are colored by magenta, while the other ToMMo individuals (463) are colored by light pink.

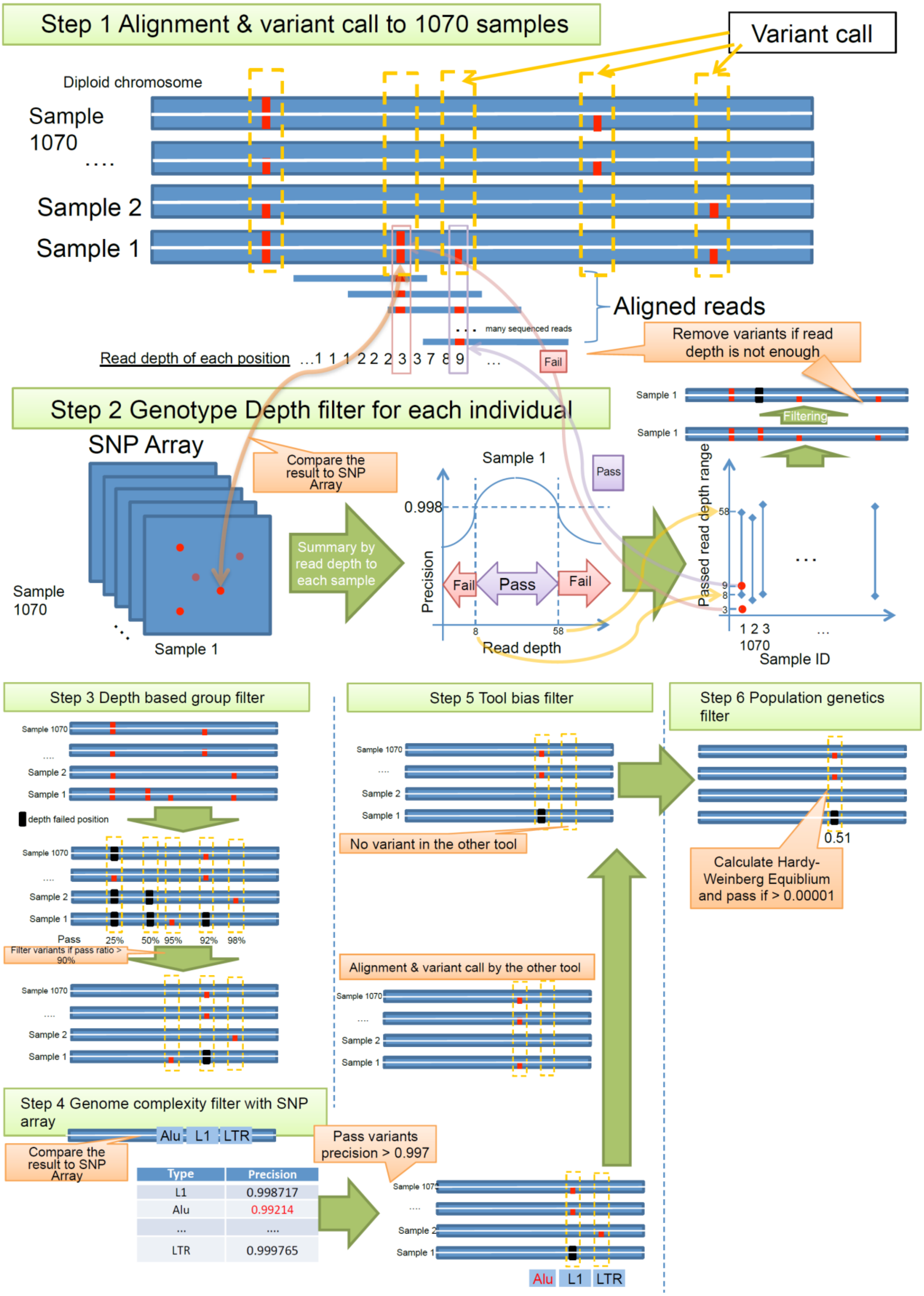

Step 1 Alignment & variant call to 1070 samples
Variant call
Diploid chromosome
Sample 1070
....
Sample 2
Sample 1
Aligned reads
many sequenced reads
Read depth of each position
...1 1 1 2 2 2 3 3 7 8 9 ...
Fail
Remove variants if read depth is not enough
Step 2 Genotype Depth filter for each individual
SNP Array
Compare the result to SNP Array
Sample 1070
Sample 1
Summary by read depth to each sample
Sample 1
Pass
0.998
Precision
Fail
Pass
Fail
8
58
Read depth
Sample 1
Filtering
Sample 1
Passed read depth range
58
9
8
3
1 2 3
...
1070
Sample ID
Step 3 Depth based group filter
Sample 1070
....
Sample 2
Sample 1
depth failed position
Sample 1070
....
Sample 2
Sample 1
Pass
25%
50%
95%
92%
98%
Filter variants if pass ratio > 90%
Sample 1070
....
Sample 2
Sample 1
Step 4 Genome complexity filter with SNP array
Alu
L1
LTR
Compare the result to SNP Array
Type | Precision
L1 | 0.998717
Alu | 0.99214
... | ....
LTR | 0.999765
Pass variants precision > 0.997
Sample 1070
....
Sample 2
Sample 1
Alu
L1
LTR
Step 5 Tool bias filter
Sample 1070
....
Sample 2
Sample 1
No variant in the other tool
Alignment & variant call by the other tool
Sample 1070
....
Sample 2
Sample 1
Step 6 Population genetics filter
0.51
Calculate Hardy-Weinberg Equiblium and pass if > 0.00001

Supplementary Figure 2. SNV Filter.

Overview of six filtering steps to obtain the high-confidence SNVs from the raw variant SNVs; Step 1: alignment and variant call to 1,070 samples, Step 2: genotype depth filter for each individual, Step 3: depth based group filter, Step 4: genome complexity filter with SNP array, Step 5: tool bias filter, and Step 6: population genetics filter. Detailed explanation for each step is described in Methods (from SNV Step 1 to SNV Step 6).

a

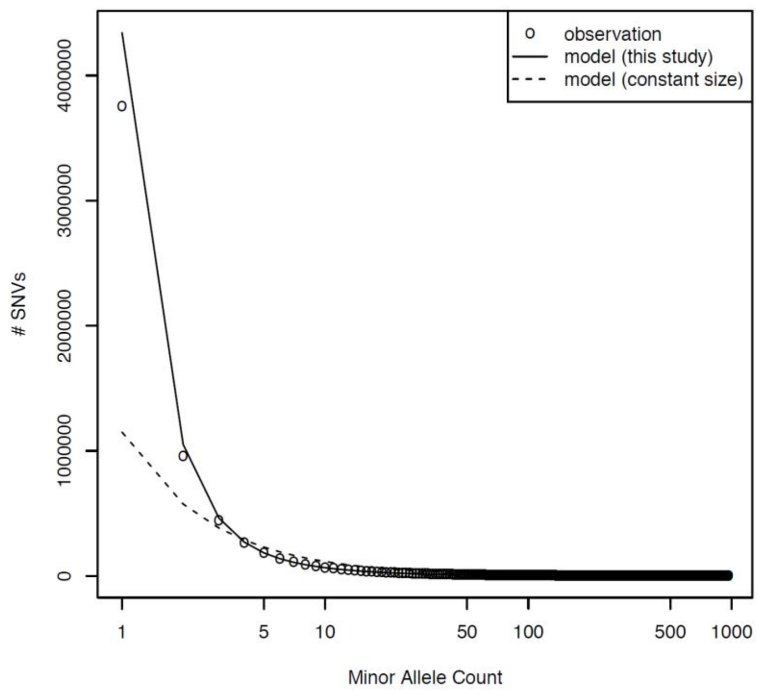

observation
model (this study)
model (constant size)
SNVs
0
1000000
2000000
3000000
4000000
1
5
10
50
100
500
1000
Minor Allele Count

b

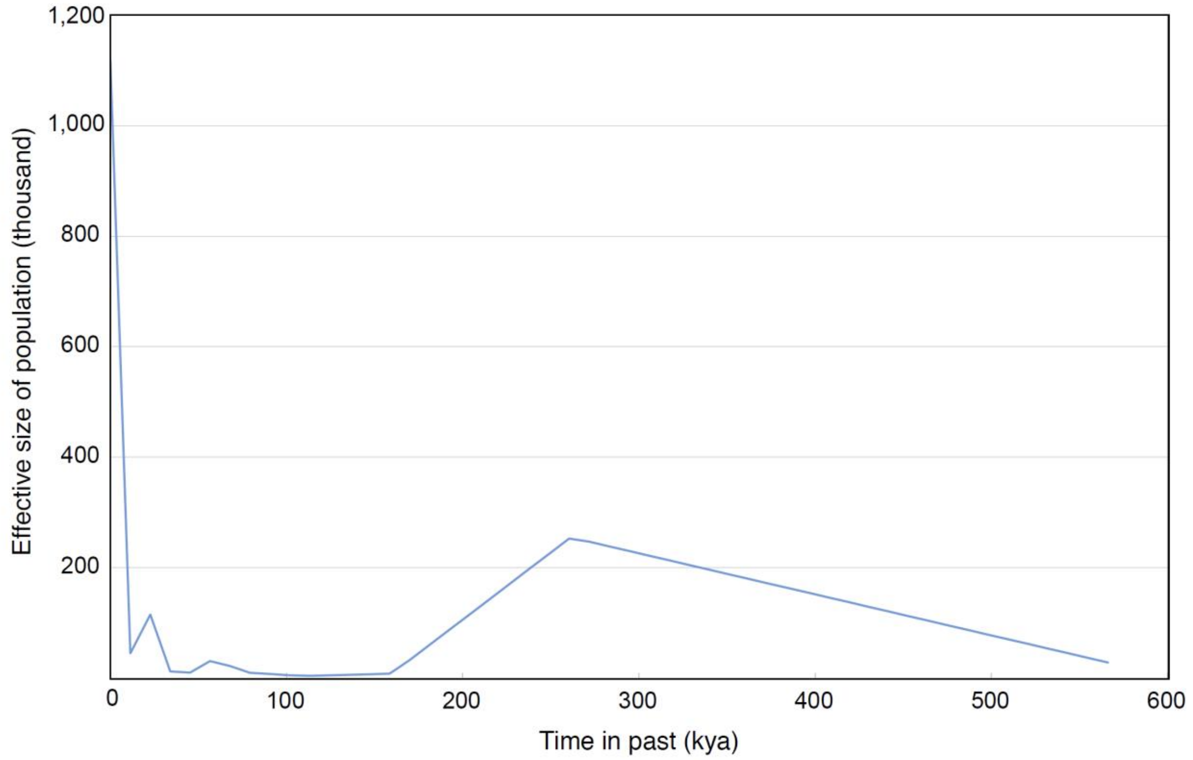

1,200
1,000
800
600
400
200
Effective size of population (thousand)
0
100
200
300
400
500
600
Time in past (kya)

Supplementary Figure 3. Demographic inference.

(a) The SFS of high-confidence SNVs from intergenic region. The simulated SFS obtained by demographic model with optimized parameters and by constant population model are shown by solid and dashed lines, respectively. (b) The change in effective population size (y-axis) is depicted against time in past (x-axis). The mutation rate and generation time are assumed to be 2.5×10^{-8} per site per generation and 25 year, respectively.

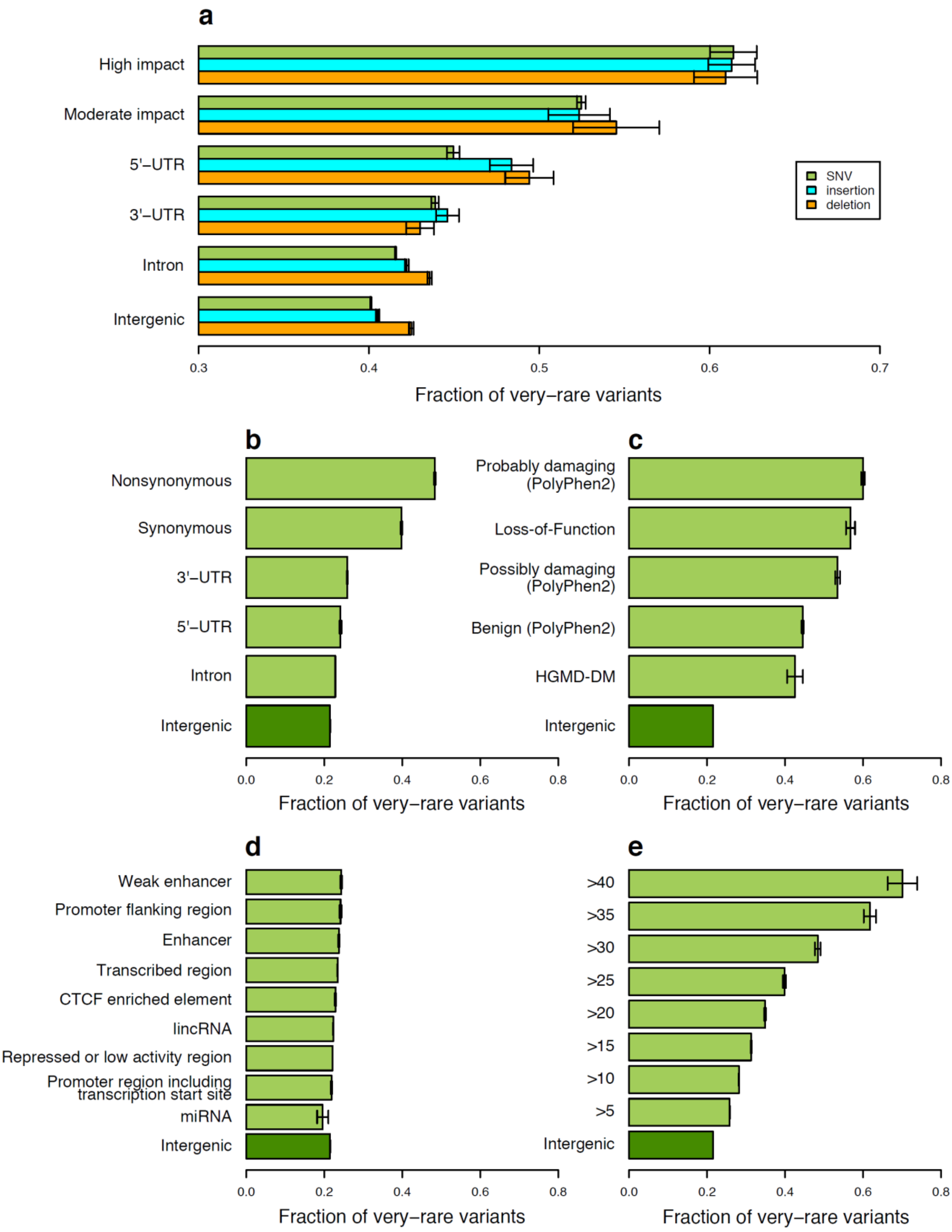
a
High impact
Moderate impact
5'–UTR
3'–UTR
Intron
Intergenic
SNV
insertion
deletion
0.3
0.4
0.5
0.6
0.7
Fraction of very–rare variants
b
Nonsynonymous
Synonymous
3'–UTR
5'–UTR
Intron
Intergenic
0.0
0.2
0.4
0.6
0.8
Fraction of very–rare variants
c
Probably damaging (PolyPhen2)
Loss-of-Function
Possibly damaging (PolyPhen2)
Benign (PolyPhen2)
HGMD-DM
Intergenic
0.0
0.2
0.4
0.6
0.8
Fraction of very–rare variants
d
Weak enhancer
Promoter flanking region
Enhancer
Transcribed region
CTCF enriched element
lincRNA
Repressed or low activity region
Promoter region including transcription start site
miRNA
Intergenic
0.0
0.2
0.4
0.6
0.8
Fraction of very–rare variants
e
>40
>35
>30
>25
>20
>15
>10
>5
Intergenic
0.0
0.2
0.4
0.6
0.8
Fraction of very–rare variants

Supplementary Figure 4. FVRV of insertions and deletions of 1KJPN and SNVs of 1KGP.

(a) The fraction of very-rare variants for the high-confidence SNVs, insertions, and deletions (≤100 bp) of 1KJPN. “High impact” and “Moderate impact” are according to the SnpEff annotation. The fraction of very-rare variants observed in the 1KGP are depicted with 95% binomial confidential interval according to (b) genomic region, (c) probable consequences for coding regions, (d) in noncoding regions, and (e) for scaled C scores. A hypergeometric projection, which subsamples each variant down to a sample size of 963 was applied to obtain the SFSs.

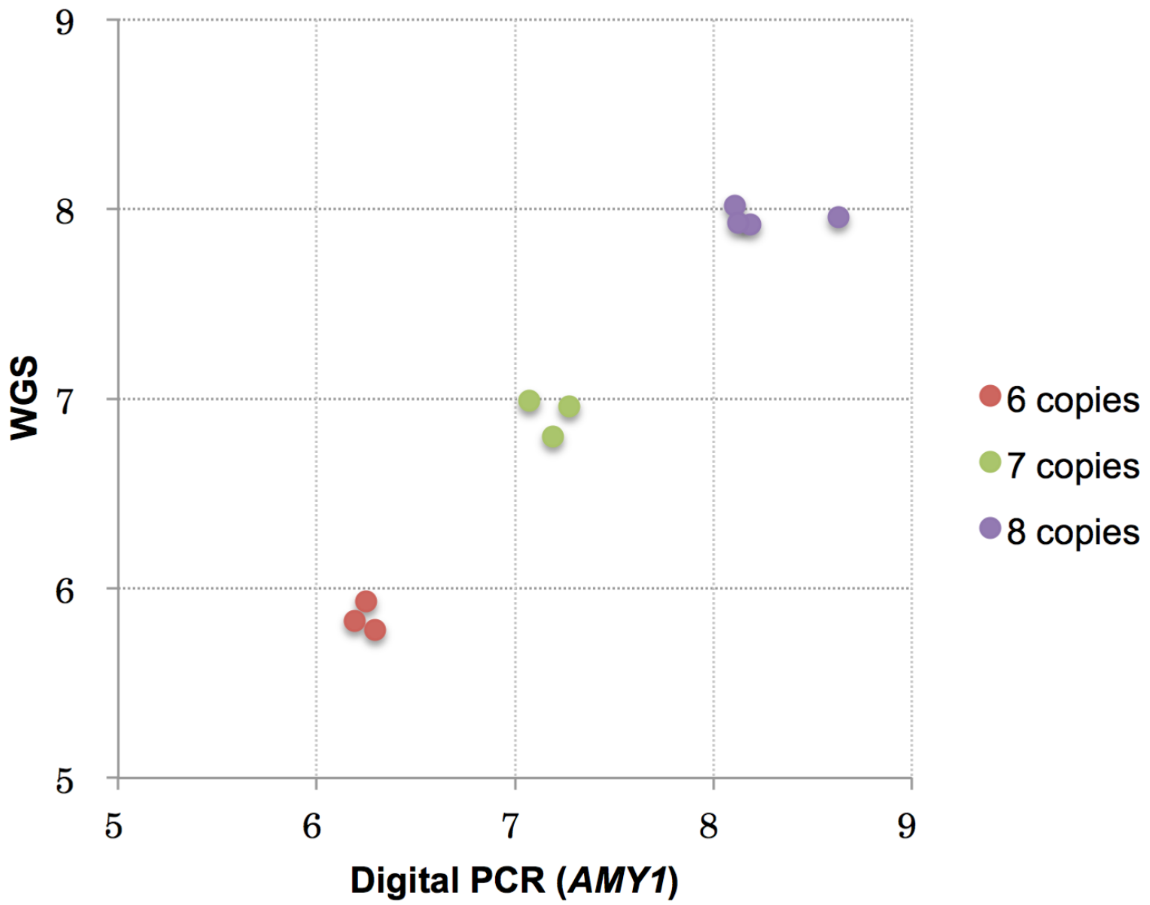

Supplementary Figure 5. Validation of diploid *AMY1* copy numbers.

The estimated diploid copy numbers of *AMY1* gene with digital PCR (in x-axis) and WGS (in y-axis). The details are described in Methods (Diploid copy numbers estimation and validation of *AMY1* genes).

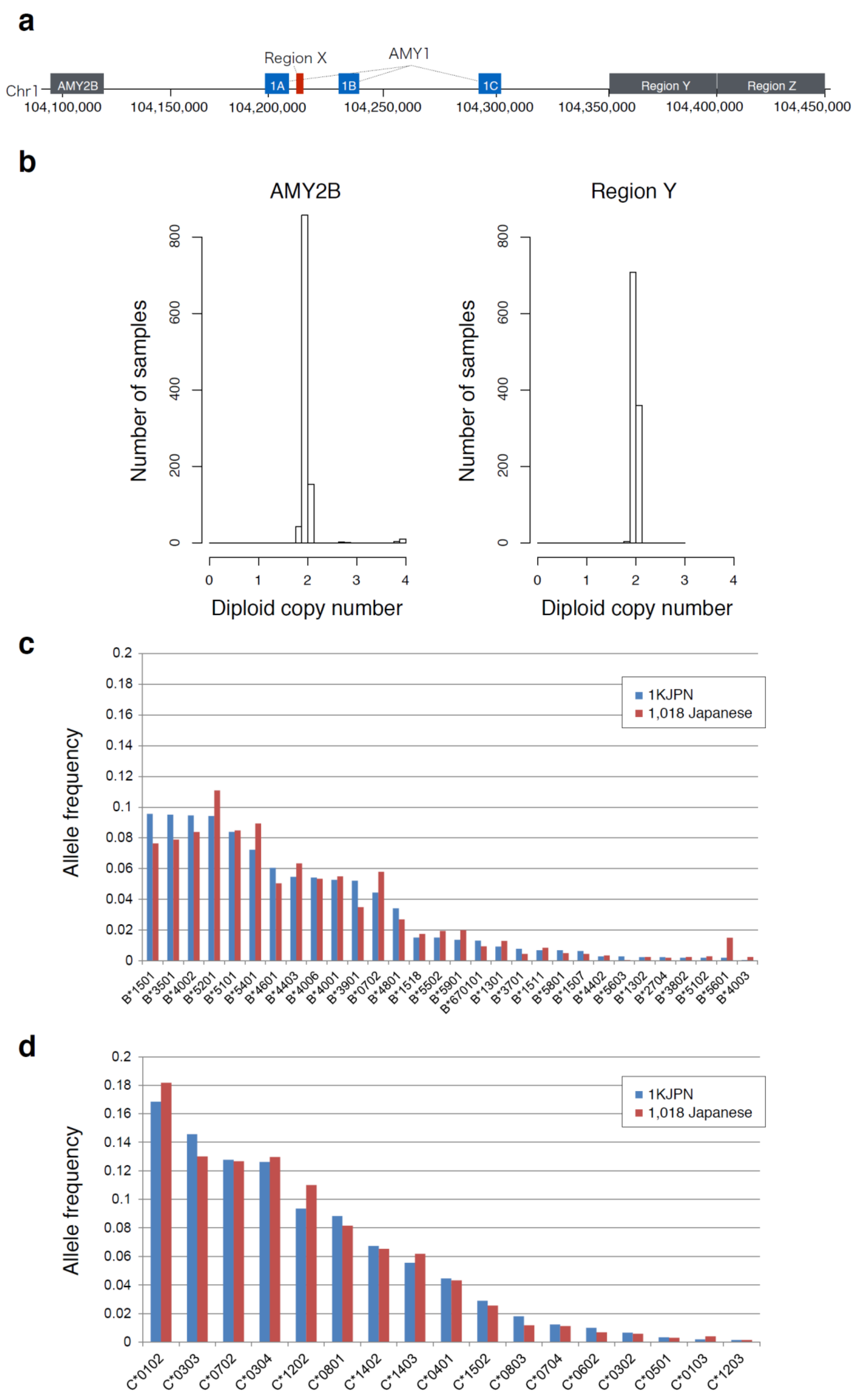
a
Region X
AMY1
Chr1
AMY2B
1A
1B
1C
Region Y
Region Z
104,100,000
104,150,000
104,200,000
104,250,000
104,300,000
104,350,000
104,400,000
104,450,000
b
AMY2B
Region Y
Number of samples
Diploid copy number
c
Allele frequency
1KJPN
1,018 Japanese
B*1501 B*3501 B*4002 B*5201 B*5101 B*5401 B*4601 B*4403 B*4006 B*4001 B*3901 B*0702 B*4801 B*1518 B*5502 B*5901 B*670101 B*1301 B*3701 B*1511 B*5801 B*1507 B*4402 B*5603 B*1302 B*2704 B*3802 B*5102 B*5601 B*4003
d
Allele frequency
1KJPN
1,018 Japanese
C*0102 C*0303 C*0702 C*0304 C*1202 C*0801 C*1402 C*1403 C*0401 C*1502 C*0803 C*0704 C*0602 C*0302 C*0501 C*0103 C*1203

Supplementary Figure 6. CNVs and HLAs.

(a) A diagram depicting the positions of *AMY2B*, *AMY1A*, Region X, *AMY1B*, *AMY1C*, Region Y, and Region Z on chromosome 1 of GRCh37 is shown. (b) Histograms of diploid copy numbers of *AMY2B* genes and Region Y. (c) Allele frequencies for *HLA-B* in 1,070 individuals in the 1KJPN estimated by high-coverage sequencing (blue), and 1,018 Japanese individuals typed by the PCR-SSOP[1] (red). (d) Allele frequencies for *HLA-C* in 1,070 individuals in the 1KJPN estimated by high-coverage sequencing (blue), and 1,018 Japanese individuals typed by the PCR-SSOP[1] (red).

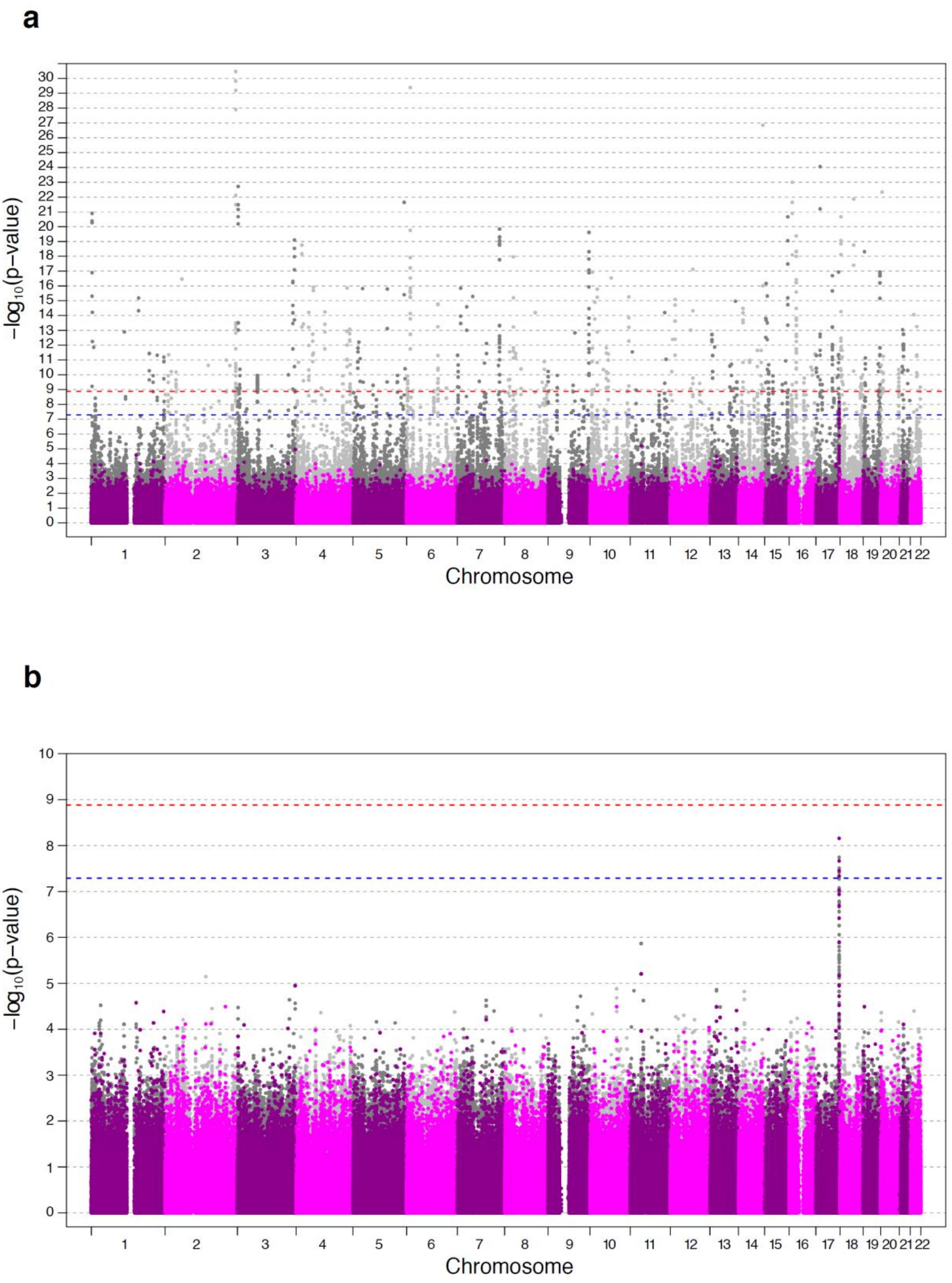
a
−log10(p−value)
30
29
28
27
26
25
24
23
22
21
20
19
18
17
16
15
14
13
12
11
10
9
8
7
6
5
4
3
2
1
0
1 2 3 4 5 6 7 8 9 10 11 12 13 14 15 16 17 18 19 20 21 22
Chromosome
b
−log10(p−value)
10
9
8
7
6
5
4
3
2
1
0
1 2 3 4 5 6 7 8 9 10 11 12 13 14 15 16 17 18 19 20 21 22
Chromosome

Supplementary Figure 7. GWAS of MMD with imputed genotypes based on 1KGP.

(a) A Manhattan plot of p-values from GWAS of the MMD with imputed genotypes based on the 1KGP reference panel of 1,092 individuals. The SNP sites from original dataset and imputed markers are plotted as dots in magenta and grey, respectively. Blue and red horizontal dotted lines display significance thresholds of the original and imputed results, respectively. (b) A Manhattan plot with imputed genotypes based on the 1KGP reference panel without 89 JPT samples.

Supplementary Table 1 Filtering of the raw SNVs to obtain the high-confidence SNVs

SNV status	Filter detail	Total	Known	Novel	Novelty rate	Pass SNVs	Removed SNVs
Raw SNVs	Raw call with bowtie2 + bcftools	27,490,104	11,914,146	15,575,958	56.66%	100.00%	-
Step 2	Filter variants in unreliable depth of coverage in the sample. (FDR<0.2%)	26,939,185	11,824,964	15,114,221	56.10%	98.00%	2.00%
Step 3	Filter variants in unreliable depth of coverage in population.	25,568,721	11,194,027	14,374,694	56.22%	93.01%	4.99%
Step 4	Filter variants categorized into low precision genomic region. (FDR <0.3%)	21,660,722	9,509,974	12,150,748	56.10%	78.79%	14.22%
Step 5	Intersect variants with other variant caller.	21,504,896	9,483,893	12,021,003	55.90%	78.23%	0.57%
Step 6	Remove SNVs with HWE < 0.00001	21,221,195	9,219,783	12,001,412	56.55%	77.20%	1.03%

SNV status corresponds to the filtering steps in Supplementary Figure 1. Total is the total number of SNVs after filtering the SNVs. Known and Novel are the known and novel SNVs in the total SNVs. The known SNVs are the SNVs reported in the dbSNP build 138. Pass SNVs are the passed SNVs from the filter.

Supplementary Table 2 FDR and CI of SNVs, deletions, and insertions in 1KJPN

SNVs					
MAF class	True	False	No call	FDR	CI
Common	40	0	22	0.00%	0.00%-4.66%
Low	76	0	48	0.00%	0.00%-2.49%
Rare and very-rare	58	0	38	0.00%	0.00%-3.24%
Total	174	0	108	0.00%	0.00%-1.10%

Deletion*					
MAF class	True	False	No call	FDR	CI
Common	23	0	8	0.00%	-
Low	7	0	5	0.00%	-
Rare and very-rare	2	0	2	0.00%	-
Total	32	0	15	0.00%	0.00%-5.78%

Insertion†					
MAF class	True	False	No call	FDR	CI
Common	18	1	5	5.26%	-
Low	2	0	0	0.00%	-
Rare and very-rare	1	0	0	0.00%	-
Total	21	1	5	4.55%	0.49%-19.34%

* Validated deletions with less than or equal to 30 bases.

† Validated insertions with less than or equal to 30 bases.

Supplementary Table 3 FDR of novel SNVs, estimated from the customized SNP array

MAF class	True	False	Validation failure*	Novelty rate in this MAF class†	FDR of known SNVs‡	Fraction of SNVs in this MAF class§	Total FDR ||	CI
Common	36	267	121	0.1%	0.03%	20.28%	0.16%	0.02%-0.30%
Low	5,446	318	333	15.2%	0.50%	12.60%	1.26%	1.03%-1.49%
Rare	7,513	90	317	61.9%	2.47%	14.82%	1.67%	1.46%-1.89%
Very-rare	7,310	33	318	87.0%	2.30%	52.31%	0.69%	0.54%-0.84%
Total FDR ||							0.80%	0.63%-0.97%

* SNVs with no call frequency > 0

† Fraction of SNVs that are not in dbSNP build 138 among the high-confidence SNVs of this MAF class

‡ Estimated from the genotyping result of HumanOmni2.5-8 BeadChip

§ Fraction of SNVs in the MAF class among the high-confidence SNVs

|| weighted average over whole SNVs

Supplementary Methods

Introduction

This section gives technical information that is not listed in the Method section of the main manuscript.

DNA preparation

Genomic DNA was extracted from buffy coats using the Gentra Puregene Blood Kit with the AutoPure LS automated DNA extraction robot (Qiagen). We followed the manufacturer's instructions, except that the RNase treatment step was omitted. The concentrations of double-stranded DNA were quantified using the PicoGreen dsDNA quantitation assay (Life Technologies), and adjusted to a concentration of 200 ng/μL with the Elution buffer (Qiagen). The DNA samples were stored at 4°C until they were used.

Sample management

The laboratory information management system (LIMS) was developed in-house and used for managing DNA samples, participant information (sex, age, and the type of blood group antigens), and genotype data (the results of sex checks and SNPs in the *ABO* gene).
The ABO blood typing for the erythrocytes from the corresponding cohort participants was performed with anti-A and anti-B monoclonal antibodies (Sysmex, Japan). The genotyping of the ABO blood type loci was done with SNPs (rs8176719 and rs8176720), which were called using whole genome sequencing (WGS) (described below) and confirmed using real-time PCR[2]. Primer sequences (5' to 3') were as follows: ABO_O-F: CCT GTG TGG ATG TGC AGT AGG A; ABO_O-R: CGT TGA GGA TGT CGA TGT TGA A; ABO_AB-Fam: 6FAM-TCC TCG TGG TGA CCC CTT GGC-BHQ1; ABO_O-Hex: 6HEX-ATG TCC TCG TGG TAC CCC TTG GCT-BHQ1; ABO_B-F: CTG CAC CTC TTG CAC CGA C; ABO_B-R: AGG CCT TCA CCT ACG AGC G; ABO_AO-Fam: 6FAM-CCC GAA GAA CCC CCC CAG GTA GTA GAA A-BHQ1; ABO_B-Hex: 6HEX-CCC GAA GAA CGC CCC CAT GTA GTA GAA A-BHQ1.

Quality control of the SNP data

One hundred and sixty nanograms of genomic DNA were analyzed with the HumanOmni2.5-8 v1.1 DNA Analysis Kit (Illumina), following the manufacturer's instructions. Briefly, genomic DNA was subjected to isothermal amplification followed by fragmentation with nucleases. The DNA was precipitated with 2-propanol, then hybridized with oligonucleotide probes immobilized on HumanOmni2.5-8 BeadChips (8 samples per BeadChip slide). After washing, probes underwent single-base extension using the captured genomic DNA as a template, and incorporating 2, 4-dinitrophenyl- or biotin-labeled nucleotides to identify the genotypes. Then, immunohistochemical staining was performed to amplify the incorporated signal. Two Robotic Universal modules (Freedom evo, TECAN, Maennedorf, Switzerland) and the Illumina Infinium LIMS system (Illumina) were used for a series of experiments. An iScan scanner system, with an AutoLoader 2.X controlled by the iScan Control Software (ver. 3.3.28: Illumina), was adopted for the data acquisition. Each SNP call was obtained using the Genotyping Module in the GenomeStudio software (ver. 2011.1: Illumina). The default set cluster file was HumanOmni2-5M-8b1-1_B.egt

(Illumina). The genotyping calls were based on a clustering algorithm, and classification was performed using the Bayesian model[3]. When the genotyping score for a SNP call was below 0.15, the corresponding locus was excluded from further analysis. After having calculated the SNP call rates, the individuals with overall call rates above 99%, and with a standard deviation of the log R ratios for the autosomal SNPs below 0.2, were used for further analysis.

Identification of cryptic relatedness in the cohort sample

The SNP-QC and the identification of cryptic relatedness were carried out with the PLINK software (ver. 1.07)[4]. We extracted SNPs using the following criteria: a Hardy–Weinberg Equilibrium test (HWET) with a p-value > 0.05, a minor allele frequency (MAF) > 0.05, and a missing data rate per locus (lmiss) < 0.01.

Closely related individuals within our cohort were identified based on an identity-by-descent (IBD) estimation. Before calculating the estimated IBD value (referred as PI-HAT in PLINK) between each pair of individuals, we carried out an LD-based pruning to extract a set of SNPs that were in nearly linkage equilibrium, using the PLINK option --indep-pairwise 200 4 0.1, as follows: SNPs within a 200-SNP window were removed one by one, until no squared correlation between the SNPs in the window was >0.1, and the procedure was iterated by sliding the window by 4 SNPs at a time. The IBDs for all pairs of individuals were estimated for the pruned SNP set using the PLINK option --genome, and individuals were removed one by one, until the PI-HAT value for no individual pair was > 0.125.

Population structure analysis

The population structure of the group comprising the selected individuals in the previous section was determined using a principal component analysis (PCA) with the smartpca program in EIGENSOFT[5]. The program was used without the auto-removal function of outlier(s) for the pruned SNP set in the previous section. Outlier individuals in the PCA were removed in the following manner: we repeatedly calculated the PCA and removed the individual with the largest absolute value of the first principal component (PC1) score, until the p-value of the Tracy-Widom statistic for the PC1 value was more than 0.05.

In addition to the above analysis, we examined the population structure of our cohort of selected individuals and HapMap reference panels[6] using a PCA to detect additional outliers. We selected SNPs that are in autosomes and satisfy the following four criteria for further analysis: polymorphic (MAF ≥ 0.05) in the ToMMo individuals, with a per-SNP call rate ≥0.95, with a HWET p-value > 0.001, and genotyped as part of the HapMap project. A PCA was separately applied to the two genotype references: the international HapMap samples and the East Asian samples (JPT and CHB). The SNP sets were pruned by LD (r^2<0.1) using PLINK, and 57,547 SNPs and 46,809 SNPs remained for these two reference panels, respectively.

We inspected the distribution of the ToMMo individuals in the two-dimensional PCA plots of the first and second PCs, respectively, and five outliers were removed (Supplementary Fig. 1). Furthermore, the population structure of the ToMMo individuals in the Japanese population was examined using a PCAj analysis (a population structure prediction system for the Japanese which is based on the largest analysis of the Japanese population structure)[7]. By using the PCAj analysis, we obtained the distribution of the ToMMo individuals by predicting the top eigenvalues for the ToMMo individuals. Using this analysis, we excluded one additional individual who was outside of the major (Hondo) cluster of the Japanese population[8].

HLA types of Japanese

The HLA loci on chromosome 6p21.3 together make up one of the most diverse and polymorphic regions in the human genome. Conventionally, HLA types have been determined at the 2-digit resolution (e.g., A*01), which approximates the serological antigen groupings. More recently, the sequence-specific oligonucleotide probes (SSOP) method has been used for HLA typing at the 4-digit resolution (e.g., A*01:01), which can distinguish amino acid differences[9]. Here, we employed a sequencing-based approach to HLA typing. Since the sequence-based approach can directly determine both coding and non-coding regions, it can achieve HLA typing at the 8-digit (e.g., A*01:01:01:01) resolution. We predicted the HLA types of the 1KJPN samples with a computational tool, HLA-VBSeq[10]. HLA-VBSeq estimates the most probable HLA alleles at full (8-digit) resolution from whole-genome sequence data. HLA-VBSeq simultaneously optimizes read alignments to the HLA allele sequences and the abundance of reads on the HLA alleles by variational Bayesian inference. HLA typing with HLA-VBSeq was carried out as follows.

First, reads obtained by whole-genome sequencing were aligned to the reference genome (GRCh37/hg19), using decoy sequences (hs37d5) with an alignment tool, BWA-MEM[11]. Second, reads aligned to HLA loci (*HLA-A, -B, -C, -DM, -DO, -DP, -DQ, -DR, -E, -F, -G, -H, -J, -K, -L, -P, -V, -MIC,* and *-TAP*) and unmapped reads were extracted from the BAM file using SAM tools[12]. If one of the paired-end mates was aligned to an HLA locus and the other was not, then both reads in the pair were extracted and used in downstream analyses. Then, the extracted reads were re-aligned to the collection of all the genomic HLA allele sequences in the IMGT/HLA database[13] (release 3.17.0), in which multiple alignments to the reference sequences for each read are allowed, with using the "-a" option in BWA-MEM. The expected read counts on the HLA alleles were estimated by variational Bayesian inference under a statistical framework. After the inference algorithm converges, the HLA types of *HLA-A*, *-B*, and *-C* loci were predicted, based on the expected number of reads assigned to each allele. A threshold for the depth of coverage of the HLA alleles was set. In our analysis, we set the threshold at a 5x depth of coverage. The details of the algorithm are described in the previous literature[10]. We compared the frequencies of HLA alleles predicted in the 1KJPN with those determined in another Japanese population with 1,018 samples[1] (Fig. 3d, Supplementary Fig. 6c, 6d). The HLA alleles that exist in at least one individual in both populations were considered for comparison.

Haplotyping of 1070 Japanese individuals

We first constructed a phased reference panel of the 1KJPN, using the SHAPEIT2[14] software (ver. 2.r644) without singletons. The high-sensitive SNVs plus the short insertions and deletions were included in the variant set. We then determined the phase of the singletons in the following manner:

(i) Locally haplotyped regions were obtained using HapMonster[15] software, which uses sequence reads spanning multiple heterozygous positions to perform local haplotyping.

(ii) If the phasing result of a locally haplotyped region from HapMonster was completely concordant with that estimated by SHAPEIT2, we added singletons in the region to the phased reference panel and phased them according to the local haplotype in the region from HapMonster. If

phasing results from SHAPEIT2 and HapMonster were discordant due to some variants in the region, singletons in the region were ignored in the phased reference panel.
(iii) Singletons not included in locally haplotyped regions were ignored in the phased reference panel. 43% of all the singletons were corrected with above procedures.

Imputation performance

We imputed the genotypes of 131 Japanese individuals not included in the 1KJPN, based on the genotypes at designed sites in the HumanOmni2.5-8 BeadChip, by using the IMPUTE2[16] software (ver. 2.2.2). The genotypes of these individuals were obtained using the same sequencing protocol and the same variant calling pipeline as were used in determining the high-sensitive SNVs set, and used as the gold standard. For the IMPUTE2 options, Ne and k_hap values were set to 20,000 and 1,000, respectively. In addition to the 1KJPN, we considered the following three reference panels for imputation, to evaluate their performance: the reference panel from the 1KGP released in Dec. 2013 and containing 1,092 cosmopolitans, a reference panel of 89 JPT individuals from the 1KGP, and a reference panel comprising both the 1KGP and the 1KJPN. To assess the agreement between the imputed genotypes and the genotype calls from NGS, we calculated the squared Pearson correlation coefficient between the gold standard genotypes, taking respective integer values of 0, 1, and 2, and values of allele dosages of imputed genotypes from 0 to 2, as in the literature[16]. Fig. 4a gives r^2 values averaged for each MAF bin size, for SNVs that exist in both the 1KJPN and the 1KGP. The MAF for each SNP was calculated independently for each reference panel. The mean r^2 value of the 1KJPN was higher than those of the other populations across the range, and the use of the ToMMo-1KGP for imputing genotypes in the Japanese population was effective, especially for low-frequency variants.

GWAS of the Moyamoya disease with imputation

We performed imputation based on a genotype dataset from a case-control study on the Japanese moyamoya disease (MMD)[17], using the reference panel of the 1KJPN. The dataset contains the genotypes of 72 Japanese MMD patients and of 45 healthy Japanese controls, from the International HapMap Project, at 1,140,419 sites designed on the Illumina HumanOmni1-Quad BeadChip. The genotypes of the case samples were obtained from the Illumina HumanOmni1-Quad BeadChip, and those of the control samples were obtained from the database of the International HapMap Project. After converting the genotype coordinate from hg18 to hg19 using liftOver, sites with a missing genotype rate > 0.01 were removed, and the remaining 975,719 sites were used in the analysis. The strand information was corrected based on the concordance of the alleles or on MAF in the 1KJPN. We performed a chi-squared test implemented in PLINK 1.07 --assoc option on these SNPs for the original and imputed datasets. For the association study on the imputed dataset, the best-guess genotypes on imputed variant positions with info metric from IMPUTE2 more than or equal to 0.5 were considered. With a significance threshold of p-value $< 5.25 \times 10^{-8}$, only a synonymous SNP, rs11870849, located at the coding region of *ENDOV* (chr17:78411073) and with a p-value of 6.95×10^{-9} was identified in the original (non-imputed) dataset (Fig. 4b). In the imputed dataset, a nonsynonymous SNP, rs112735431, located in RNA213 (chr17:78358945), was identified as having the highest SNP association with a p-value of 8.07×10^{-10}, which is beyond the significance threshold, at a p-value $< 5.06 \times 10^{-9}$ (Fig. 4d). We also applied the reference panel from the 1KGP to the MMD dataset for the imputation. A Manhattan plot from the imputed genotypes appeared to

contain many spurious associations with MMD (Supplementary Fig. 7a). Since the control samples of the MMD dataset are also included in 1KGP as HapMap JPT samples, we performed association studies for genotypes imputed with the panel without HapMap JPT samples. Supplementary Fig. 7b shows a Manhattan plot from the imputed genotypes, where rs112735431 was not detected as a significant variant.

Individual mutation load

We identified overlaps between the high-confidence SNVs and known pathological SNVs from the Human Gene Mutation Database (HGMD)[18]. Possible pathological SNVs were identified based on the genomic coordinates and on the consistency of the allele bases. After this filtering step, 4,368 pathological SNVs in the HGMD, including 1,002 disease-causing mutations (DMs), were extracted. Stop-gained alleles were annotated using the SnpEff software (ver. 3.3c). For each individual, the number of these functional variants (HGMD-DM and stop-gained alleles) was counted for each variant class. We used sites of functional variants that satisfy the following conditions; (i) the ancestral states were inferred with high-confidence, (ii) the functional variants were non-reference (alternative) alleles, and (iii) the functional variants were derived alleles. We discarded stop-gained SNVs if the proportion of truncated ORFs was less than 5%.

Variants discovery rate

Although the variants discovery rate can be predicted from preliminary data[19,20], we estimated the discovery rate using variants data obtained in this study. Let us consider a sampling probability of variants $P(n, q_{min})$ from a population with the allele frequency distribution $F(q)$ where q is an allele frequency of a variants in very large population. In a sample of n individuals comprising $2n$ chromosomes from the population, the sampling probability of variants with minor allele frequency equal or greater than $q_{\min}$ can be written as follows:

$$P(n, q_{\min}) = \frac{\sum_{k=1}^{2n-1} \int_{q_{\min}}^{1-q_{\min}} \binom{2n}{k} q^k (1-q)^{2n-k} F(q) dq}{\sum_{k=0}^{2n} \int_{q_{\min}}^{1-q_{\min}} \binom{2n}{k} q^k (1-q)^{2n-k} F(q) dq} = \frac{\int_{q_{\min}}^{1-q_{\min}} \{1-(1-q)^{2n}\} F(q) dq}{\int_{q_{\min}}^{1-q_{\min}} F(q) dq} \qquad (1)$$

Demographic inference

In a constant population size, the allele frequency distribution under the equilibrium state is

$$F(q) \propto \frac{1}{q(1-q)}.$$

However, such setting is not realistic for human population where the complex demographic events such as recent size expansion and bottleneck have been experienced. Thus, we inferred the demographic history of the 1KJPN was inferred using a forward-time simulation under the Wright-Fisher diffusion model[21] (Supplementary Fig. 3). The time-course of the allele frequency (q) distribution in a population, $f(q, t)$, is approximated by solving the following diffusion equation:

$$\frac{\partial f(q,t)}{\partial t} = \frac{1}{2} \frac{\partial^2}{\partial t^2} \{V(q) f(q,t)\} - \frac{\partial}{\partial t} \{M(q) f(q,t)\}, \qquad (2)$$

where $M(q)$ and $V(q)$ represent the mean and variance of the change in the allele frequency at frequency q, respectively. Consider a variable population size with additive selection in the Wright-Fisher model, and let $\rho(t)$ and N_0 be the relative population size and the effective population size at time 0, respectively, $V(q)$ and $M(q)$ are respectively written as follows:

$$V(q) = \frac{q(1-q)}{2N_0\rho(t)}$$

$$M(q) = sq(1-q),$$

where s is an advantageous selection coefficient. The time unit of Equation (2) can be converted into a unit of $2N_0$ generations:

$$\frac{\partial f(q,T)}{\partial T} = \frac{1}{2}\frac{\partial^2}{\partial T^2}\left\{\frac{q(1-q)f(q,T)}{\rho(T;\boldsymbol{\lambda})}\right\} - \frac{\partial}{\partial T}\{Sq(1-q)f(q,T)\} \quad (3)$$

where S=$2N_0s$ and T=$2N_0t$. Equation (3) was solved numerically with uneven grid spacing, as previously described[22]. The changes in the population size are represented by a series of linear equations, whose tips are connected each other.

$$\rho(T;\boldsymbol{\lambda}) = \frac{\lambda_{i+1}-\lambda_i}{m_{i+1}-m_i}T + \lambda_i - \frac{\lambda_{i+1}-\lambda_i}{m_{i+1}-m_i}m_i$$

$$m_{i+1} \le T < m_i,$$

where m_i is the time in units of $2N_0$, and λ_i is the relative population size at time m_i. Time intervals between m_i and m_{i+1} was set to be proportional to $1/i(i+1)$ so that the time interval become shorter as time is closer to the present time. The expected number of sites with an i derived allele in a sample size n, i.e., the site frequency spectrum (SFS), is calculated using the following equation:

$$g(i,n;\lambda) = \theta\int_0^1 \binom{n}{i} q^i(1-q)^{n-i} f(q,T;\boldsymbol{\lambda})dq,$$

where θ=$2N_0U$ and U is the mutation rate for a set of sites of interest (e.g. intergenic).
Let $\mathbf{X}=(x_1, x_2, \ldots, x_i, \ldots, x_{n/2})$ be the observed number of sites with a minor allele count i. Assuming that each x follows an independent Poisson distribution, the likelihood function with folded SFS X is written as the following equation.

$$L(\boldsymbol{\lambda};\mathbf{X}) = \prod_{i=1}^{n}\frac{e^{-\{g(n,i;\boldsymbol{\lambda})+g(n,n-i;\boldsymbol{\lambda})\}}\{g(n,i;\boldsymbol{\lambda})+g(n,n-i;\boldsymbol{\lambda})\}^{x_i}}{x_i!}$$

The m_is and time of simulation τ were set a priori, and the λ_i and θ values were estimated by maximizing likelihood function $L(\lambda:\mathbf{X})$, with the L-BFGS-B algorithm[23]. Applying the maximum likelihood estimates of demographic parameter $\hat{\boldsymbol{\lambda}}$, the allele frequency distribution $F(q)$ in Equation (1) can be obtained as follows:

$$F(q) = f(q,T;\hat{\boldsymbol{\lambda}})$$

The demographic parameter $\hat{\boldsymbol{\lambda}}$ was estimated from the SFS of the high-confidence SNVs of intergenic regions (Supplementary Fig. 3a) and the number of time intervals was set to be 12.

The inferred demographic model was shown in Supplementary Fig. 3b. The variants discovery rate in Fig. 1d was estimated according to Equation (1) under this model.

Supplementary References

1. Itoh Y, *et al.* High-throughput DNA typing of HLA-A, -B, -C, and -DRB1 loci by a PCR-SSOP-Luminex method in the Japanese population. *Immunogenetics* **57**, 717-729 (2005).

2. Ogata S, Katagiri H, Kobayashi M, Ui H, Yoshii T. An examination by TaqMan PCR method with a specific probe on ABO blood typing. *Japanese J Forensic Sci and Tech* **12**, 167-176 (2007).

3. Oliphant A, Barker DL, Stuelpnagel JR, Chee MS. BeadArray technology: enabling an accurate, cost-effective approach to high-throughput genotyping. *BioTechniques* **Suppl**, 56-58, 60-51 (2002).

4. Purcell S*, et al.* PLINK: a tool set for whole-genome association and population-based linkage analyses. *Am J Hum Genet* **81**, 559-575 (2007).

5. Patterson N, Price AL, Reich D. Population structure and eigenanalysis. *PLoS Genet* **2**, e190 (2006).

6. International HapMap Consortium. Integrating common and rare genetic variation in diverse human populations. *Nature* **467**, 52-58 (2010).

7. Kumasaka N, Yamaguchi-Kabata Y, Takahashi A, Kubo M, Nakamura Y, Kamatani N. Establishment of a standardized system to perform population structure analyses with limited sample size or with different sets of SNP genotypes. *J Hum Genet* **55**, 525-533 (2010).

8. Yamaguchi-Kabata Y*, et al.* Japanese population structure, based on SNP genotypes from 7003 individuals compared to other ethnic groups: effects on population-based association studies. *Am J Hum Genet* **83**, 445-456 (2008).

9. Levine JE, Yang SY. SSOP typing of the Tenth International Histocompatibility Workshop reference cell lines for HLA-C alleles. *Tissue Antigens* **44**, 174-183 (1994).

10. Nariai N*, et al.* HLA-VBSeq: accurate HLA typing at full resolution from whole-genome sequencing data. *BMC Genomics* **16 Suppl 2**, S7 (2015).

11. Li H, Durbin R. Fast and accurate short read alignment with Burrows-Wheeler transform. *Bioinformatics* **25**, 1754 - 1760 (2009).

12. Li H*, et al.* The Sequence Alignment/Map format and SAMtools. *Bioinformatics* **25**, 2078-2079 (2009).

13. Robinson J, Halliwell JA, McWilliam H, Lopez R, Parham P, Marsh SG. The IMGT/HLA database. *Nucleic Acids Res* **41**, D1222-1227 (2013).

14. Delaneau O, Zagury J-F, Marchini J. Improved whole-chromosome phasing for disease and population genetic studies. *Nat Methods* **10**, 5-6 (2013).

15. Kojima K*, et al.* HapMonster: A Statistically Unified Approach for Variant Calling and Haplotyping Based on Phase-Informative Reads. *Lecture Notes in Comput. Sci.* **8542**, 107-118 (2014).

16. Howie BN, Donnelly P, Marchini J. A flexible and accurate genotype imputation method for the next generation of genome-wide association studies. *PLoS Genet* **5**, e1000529 (2009).

17. Kamada F*, et al.* A genome-wide association study identifies RNF213 as the first Moyamoya disease gene. *J Hum Genet* **56**, 34-40 (2011).

18. Cooper D. The human gene mutation database. *Nucleic Acids Res* **26**, 285-287 (1998).

19. Ionita-Laza, I., Lange, C. & N, M. L. Estimating the number of unseen variants in the human genome. *Proc Natl Acad Sci U S A* **106**, 5008-5013 (2009).

20. Gravel, S., National Heart, Lung, and Blood Institute (NHLBI) GO Exome Sequencing Project, Predicting discovery rates of genomic features. *Genetics* **197**, 601-610 (2014).

21. Crow JF, Kimura M. An introduction to population genetics theory. *An introduction to population genetics theory* (1970).

22. Evans SN, Shvets Y, Slatkin M. Non-equilibrium theory of the allele frequency spectrum. *Theor Popul Biol* **71**, 109-119 (2007).

23. Byrd RH, Lu P, Nocedal J, Zhu C. A limited memory algorithm for bound constrained optimization. *SIAM J Sci Comput* **16**, 1190-1208 (1995).
